# Spectrum of Primary Hemophagocytic Lymphohistiocytosis‐Associated Gene Mutations in Chinese Patients

**DOI:** 10.1002/mco2.70979

**Published:** 2026-09-02

**Authors:** Wenshuai Zheng, Shenyu Wang, Zhenlan Du, Lianming Liao, Xiaohong Li, Hongmei Ning

**Affiliations:** ^1^ Department of Hematology Hainan Hospital of Chinese PLA General Hospital Sanya Hainan China; ^2^ Senior Department of Hematology Chinese PLA General Hospital Beijing China; ^3^ Senior Department of Pediatric Chinese PLA General Hospital Beijing China; ^4^ Department of Laboratory Medicine Fujian Medical University Union Hospital Fuzhou China

**Keywords:** China, epidemiology, hemophagocytic lymphohistiocytosis, multicenter, mutation spectrum

## Abstract

Hemophagocytic lymphohistiocytosis (HLH) is a life‐threatening disease characterized by hyperinflammation. Primary HLH (primary HLH), resulting from genetic mutations, is a subtype of HLH. The mutation spectrum of primary HLH‐associated genes has not been well determined in China. This study aimed to explore the mutation spectrum of 12 primary HLH‐associated genes in a multicenter database. Medical records and gene sequencing data of 1224 HLH patients were retrieved from January 2014 to September 2024, and gene mutations were analyzed. Genetic variants were observed in 350 (28.59%) patients. Among them, 108 patients had a definitive genetic diagnosis, 227 patients carried single heterozygous variants, and 15 patients carried digenic/polygenic heterozygous variants. A total of 275 different variants were identified, and missense variants were most common. Variants in *UNC13D* were most common, followed by *LYST* and *PRF1*, while variants in *MAGT1*, *ITK*, and *CD27* were rare. For *UNC13D, LYST*, and *PRF1*, variants c.2588G>A (p.Gly863Asp), c.368A>G (p.His123Arg), and c.1349C>T (p.Thr450Met) were most common, respectively. We described the mutation spectrum of primary HLH‐associated genes in a largest Chinese cohort and found some mutation specificity for Chinese HLH patients. Our data might help design sequencing panels, interpret sequencing results, and understand genetic background of Chinese HLH patients.

## Graphical abstract

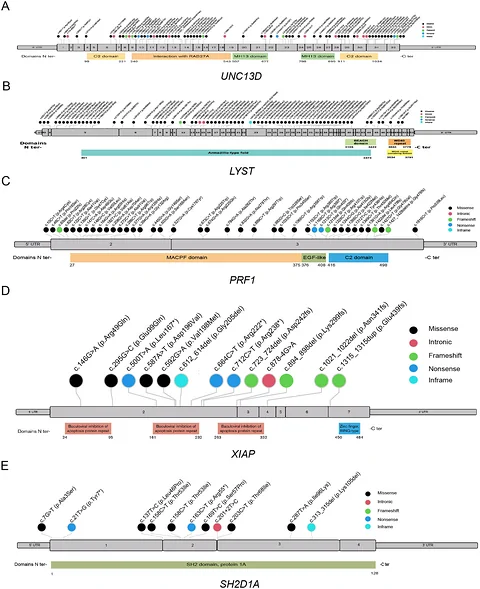

## 1 Background

Hemophagocytic lymphohistiocytosis (HLH) is an aggressive and life‐threatening immunological syndrome characterized by abnormal activation of cytotoxic T lymphocytes, natural killer (NK) cells, and macrophages, resulting in cytokine‐mediated tissue injury and multiorgan dysfunction. HLH is classified as primary HLH and secondary HLH according to the etiology [1].

Primary HLH, predominantly affecting children, results from inherited immune defects, typically involving impaired cytotoxic lymphocyte function (NK cells and cytotoxic T cells) due to mutations disrupting the perforin‐granzyme cytolytic pathway. Secondary HLH, more common in adults, is often triggered by infections, malignancies, or autoimmune diseases, usually in the absence of known primary HLH genetic defects or family history [2]. The early differentiation between primary HLH and secondary HLH has important clinical implications. Although aggressive immunosuppressive therapy is required for both primary HLH and secondary HLH in the initial period, the post‐remission management of primary HLH and secondary HLH is variable [3]. For primary HLH, allogeneic hematopoietic stem cell transplantation should be considered speedily, while the treatment should be focused on eliminating the secondary triggers for secondary HLH [3].

In the past, diagnosis of patients with primary HLH was based on an early age at onset of disease and a positive family history. However, with the development of gene sequencing technology, identification of the genetic defects in HLH has made the diagnosis of primary HLH more accurate, and new primary HLH‐associated genes are constantly being discovered. Presently, more than 100 HLH‐associated genes have been reported. Classic genetic diseases with HLH as the predominant manifestation include family HLH (FHL), primary immunodeficiency syndromes (PID)‐associated HLH, X‑linked lymphoproliferative disease (XLP), and Epstein–Barr virus (EBV)‐driven HLH [4]. These genetic mutations may impact cytolytic functions, lymphocyte survival, and inflammasome activation. FHL is the most common causes of primary HLH and is subclassified into five types based on the underlying genetic mutations. The etiology of FHL type 1 remains to be elucidated [5]. FHL types 2–5 are caused by mutations in *PRF1*, *UNC13D*, *STX11*, and *STXBP2*, respectively [6, 7, 8, 9]. PID‐associated HLH mainly include Chediak–Higashi syndrome, Griscelli syndrome type 2, and Hermansky–Pudlak syndrome type 2, which are caused by mutations in *LYST*, *RAB27A*, and *AP3B1*, respectively [10, 11, 12]. XLP, mainly including XLP‐1 and XLP‐2, is caused by mutations in *SH2D1A* and *XIAP*, respectively [13, 14]. For EBV‐driven HLH, mutations in *MAGT1*, *ITK*, *CD27*, *CD70*, *CTPS1*, and *RASGRP1* have been reported [4]. Therefore, many genes are involved in the pathogenesis of HLH.

Gene mutations in primary HLH vary significantly in different ethnic groups [15, 16, 17]. For example, *PRF1* is the most commonly mutated gene in European and American populations while the most commonly mutated gene in Korean population is *UNC13D*. Understanding the pattern of primary HLH‐associated gene mutations in a particular population might help design cost‐effective mutation screening strategies and interpret the sequencing results. Although China has a large population, available data on primary HLH‐associated gene mutations in Chinese population are limited [17, 18, 19, 20]. The largest study only included 311 HLH patients [18]. Therefore, we performed this multicenter study to analyze genetic variants in clinically confirmed or suspected HLH patients who underwent targeted next‐generation sequencing (t‐NGS).

## 2 Results

### 2.1 Patients’ characteristics

A total of 1224 patients were included. The median age of onset was 24 years (IQR, 4–45) and 55.40% of the patients were older than 18 years of age. Among them, 350 (28.59%) possessed primary HLH‐associated gene variants, and 202 (57.71%) were male. The characteristics of 350 patients can be seen in Table 1. A total of 108 patients carried a definite genetic diagnosis, including homozygous variants (*n* = 24), compound heterozygous variants (*n* = 55), and X‐linked hemizygous variants (*n* = 29); 227 patients carried single heterozygous variants; and 15 patients carried digenic/polygenic heterozygous variants. Compared with patients with definitive genetic diagnosis (median age: 10 years [IQR, 2–27.3]), Mann–Whitney *U* test showed that patients with single heterozygous variants (median age: 21.5 years [IQR, 4–40]) had significantly older age of onset (*p* = 0.002) and the age of onset of patents with digenic/polygenic heterozygous variants (median age: 17 years [IQR, 2.6–42.5]) was not significantly different (*p* = 0.433). The detailed genetic variants are listed in Tables S1–S3.

**TABLE 1:** The characteristics of patients with HLH‐associated gene variants.

Characteristics	Patients with definitive genetic diagnosis	Patients with single heterozygous variants	Patients with digenic/polygenic variants
Age (years, median and IQR)	10 (2‐27.3)	21.5 (4‐40)	17 (2.6‐42.5)
Sex (male/female)	76/32	123/104	3/12
Number of patients with different gene variants			
UNC13D	30 (9 Hom/21 Com)	79	9
PFR1	30 (12 Hom/18 Com)	28	0
LYST	14 (Com)	63	11
STXBP2	2 (Com)	23	3
AP3B1	0	14	2
XIAP	13 (Hemi)	2	0
SH2D1A	13 (Hemi)	1	0
RAB27A	2 (1 Hom/1 Com)	6	5
STX11	0	8	1
MAGT1	3 (Hemi)	0	0
ITK	1 (Hom)	2	0
CD27	0	1	0

Abbreviations: Com, compound heterozygous; Hemi, hemizygous; HLH, hemophagocytic lymphohistiocytosis; Hom, homozygous; IQR, interquartile ranges.

### 2.2 Analysis of Variants

**FIGURE 1:**
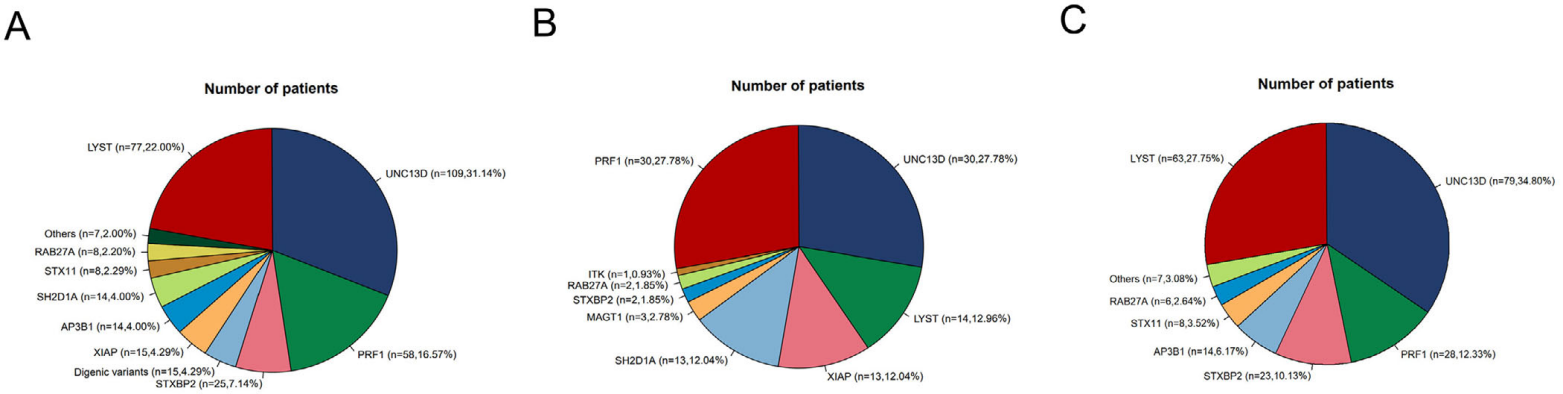
Proportion of patients with primary HLH‐associated gene variants in (A) all patients, (B) patients with definite genetic diagnosis, and (C) patients with single heterozygous variant.

A total of 445 mutant alleles and 275 different variants were identified in 350 patients. Of these patients, 4.96% (15/350) of patients had digenic/polygenic heterozygous variants. In addition, the majority (200/350, 57.14%) carried FHL‐associated gene variants: 109 (31.14%) with variants in *UNC13D*, 58 (16.57%) with variants in *PRF1*, 25 (7.14%) with variants in *STXBP2*, and 8 (2.29%) with variants in *STX11*. Patients with PID‐associated gene variants accounted for 28.29% (99/350) of all patients: 77 (22.00%) with variants in *LYST*, 14 (4.00%) with variants in *AP3B1*, and 8 (2.29%) with variants in *RAB27A*. Patients with X‐linked variants accounted for 9.14% (32/350) of all patients: 15 (4.29%) with variants in *XIAP*, 14 (4.00%) with variants in *SH2D1A*, and 3 (0.86%) with variants in *MAGT1*. The proportion of patients with different primary HLH‐associated gene variants in different groups is shown in Figure 1. Unexpectedly, variants in *STX11*, *AP3B1*, and *CD27* were not found in patients with a definitive genetic diagnosis.

**FIGURE 2:**
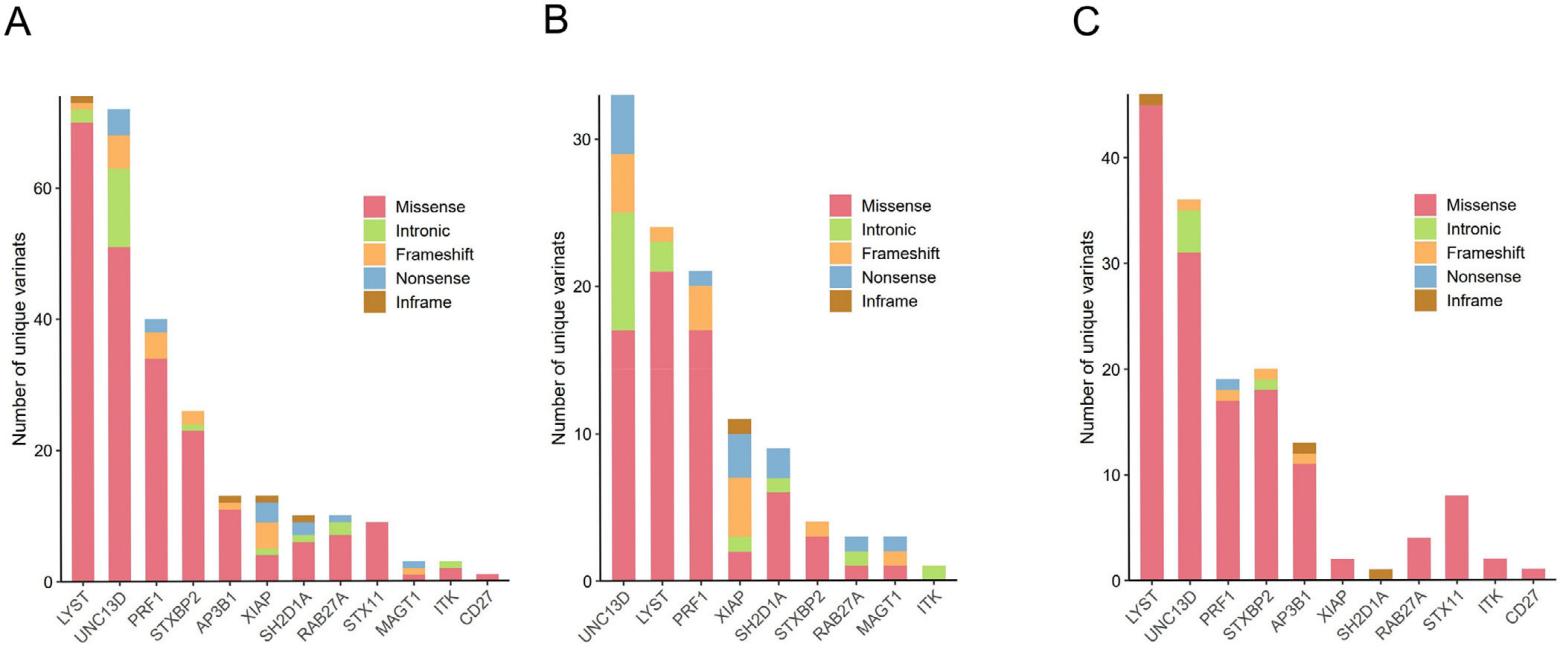
Distribution of mutated type of primary HLH‐associated genes in (A) all patients, (B) patients with definite genetic diagnosis, and (C) patients with single heterozygous variant.

In 275 different variants, including 29 pathogenic ones, 28 likely pathogenic ones and 218 variants of uncertain significance, missense variants (79.63%, 219/275) were most common, followed by intronic variants (7.27%, 20/275), frameshift variants (6.91%, 19/275), and nonsense variants (4.73%, 13/275), while inframe variants (1.45%, 4/275) were rare. The distribution of mutated type of primary HLH‐associated genes in different groups is shown in Figure 2.

### 2.3 Mutation Spectrum Analysis

**FIGURE 3:**
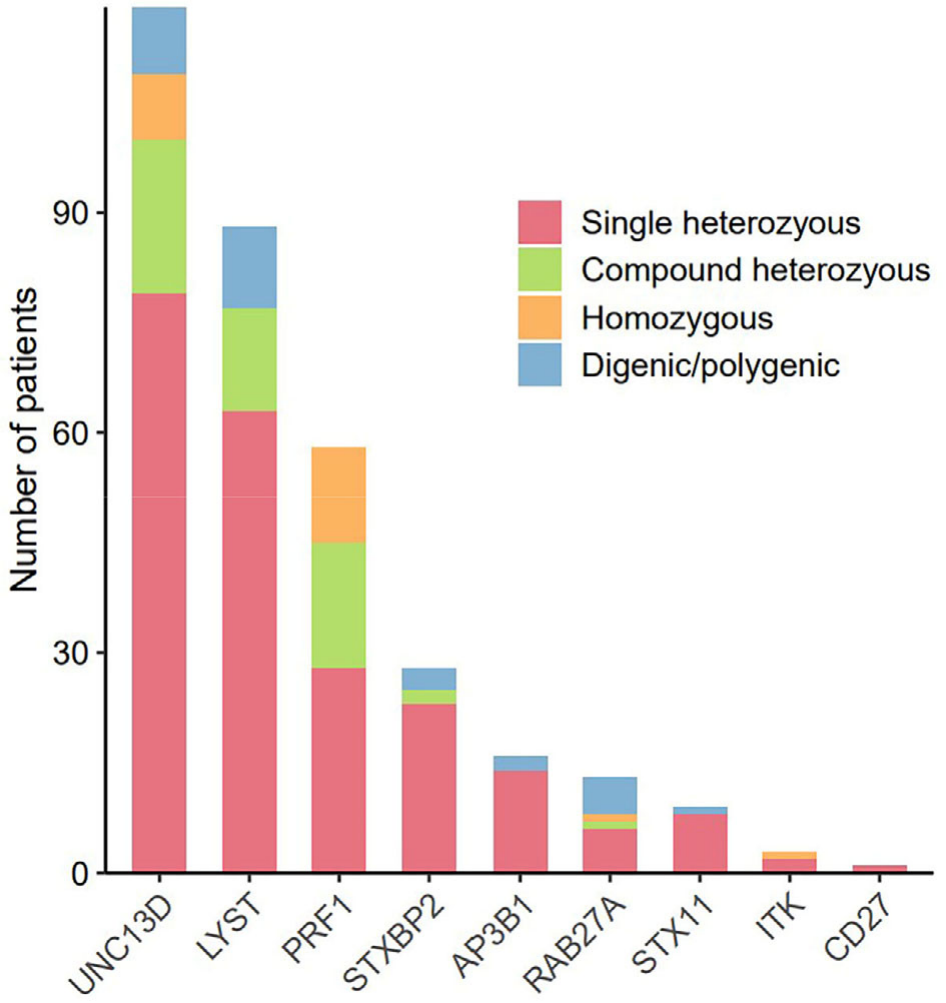
Distribution of different type of zygote of primary HLH‐associated genes.

**FIGURE 4:**
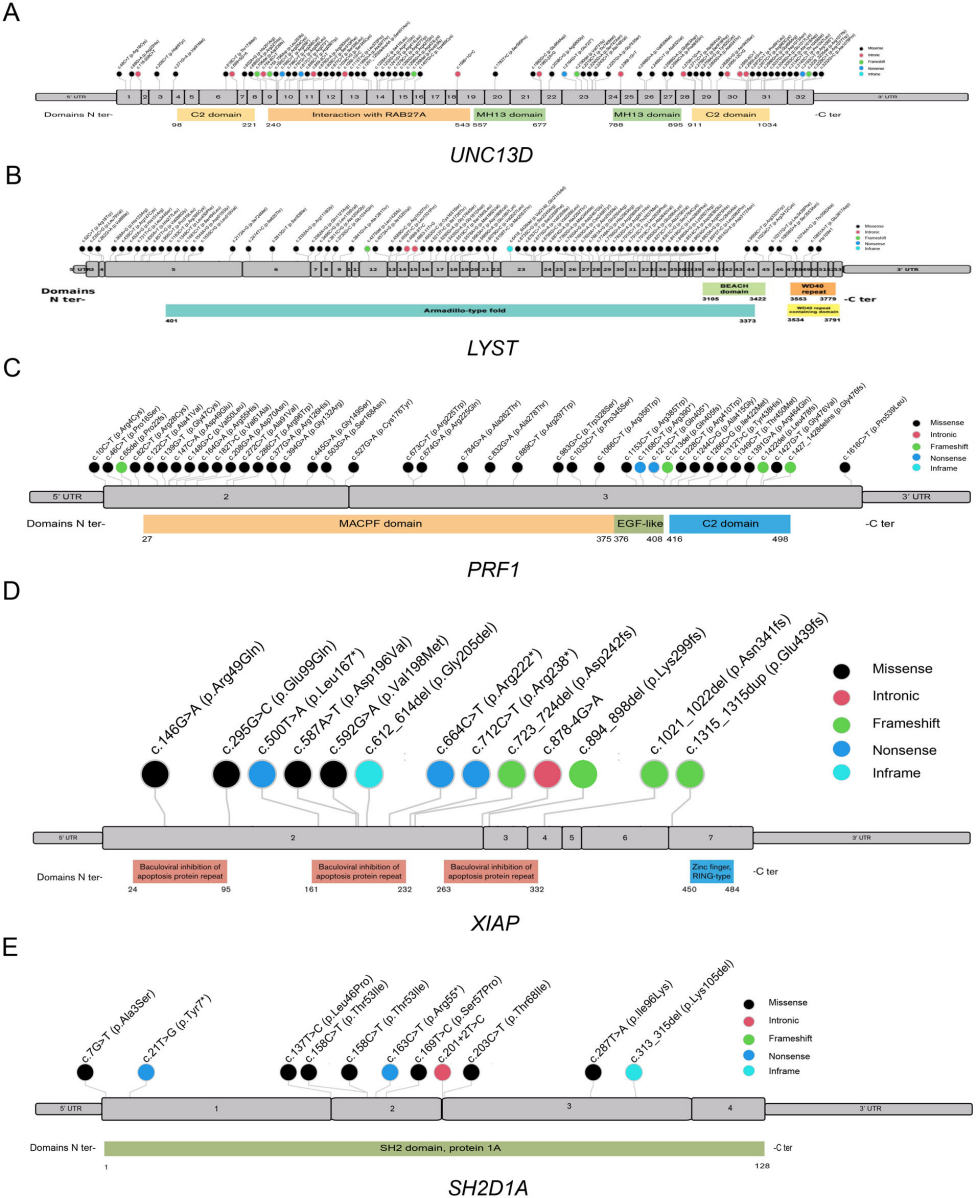
The locations and types of variants in the most frequently affected genes. (A) A total of 73 different variants of UNC13D were identified in 118 patients. (B) A total of 74 different variants of LYST were identified in 88 patients. (C) A total of 40 different variants of PRF1 were identified in 58 patients. (D) A total of 13 different variants of XIAP were identified in 15 patients. (E) A total of 10 different variants of SH2D1A were identified in 14 patients. The gray rectangles represent the respective exons. Functional domains are color‐coded and numbers below correlate to the amino acid sequence. Each circle represents a detected variant, and the different colors of the circles depict different mutant types.

In our cohort, *UNC13D* was the most frequently mutated gene, with 148 mutant alleles (73 unique variants) identified in 118 patients, including 9 homozygotes, 21 compound heterozygotes, 79 single heterozygotes, and 9 digenic/polygenic heterozygotes. The distribution of different types of zygote of primary HLH‐associated genes are demonstrated in Figure 3. Variant c.2588G>A (p.Gly863Asp) was the most common variant in *UNC13D*, appearing in 39 alleles in 33 patients, including 6 homozygotes, 4 compound heterozygotes, 22 single heterozygotes, and 1 digenic/polygenic heterozygotes. The other two common variants were c.1228A>C (p.Ile410Leu) and c.602A>G (p.His201Arg), accounting for nine alleles in nine patients and five alleles in five patients, respectively (Tables S1–S3; Figure 4A).

*LYST* was the second most frequently mutated gene, with 102 mutant alleles (74 unique variants) identified in 88 patients, including 14 compound heterozygotes, 63 single heterozygotes, and 11 digenic/polygenic heterozygotes. The most frequent variant was c.368A>G (p.His123Arg), appearing in six alleles in six patients, including one compound heterozygote, four single heterozygotes, and one digenic/polygenic heterozygotes (Tables S1–S3; Figure 4B).

*PRF1* was the third most frequently mutated gene, with 88 mutant alleles (40 unique variants) identified in 58 patients, including 13 homozygotes, 17 compound heterozygotes, and 28 single heterozygotes. The most frequent variant in *PRF1* was c.1349C>T (p.Thr450Met), appearing in 21 alleles in 13 patients, including eight homozygotes, one compound heterozygotes, and four single heterozygotes. The other two common variants were c.503G>A (p.Ser168Asn) and c.65del (p.Pro22fs), accounting for nine alleles in nine patients and seven alleles in seven patients, respectively. In addition, five patients were found to have compound heterozygotes, including c.65del (p.Pro22fs) and c.503G>A (p.Ser168Asn) variants (Tables S1–S3; Figure 4C).

Twenty‐six different variants in *STXBP2* were identified in 31 alleles in 29 patients, including two compound heterozygotes, 23 single heterozygotes, and four digenic/polygenic heterozygotes. Nine different variants in *STX11* identified nine alleles in nine patients, including eight heterozygotes and one digenic/polygenic heterozygote. There were no repetitive variants in *STXBP2* and *STX11* (Tables S1–S3).

Ten different variants in *RAB27A* were identified in 14 alleles in 13 patients, including one homozygote, one compound heterozygote, six single heterozygotes, and five digenic/polygenic heterozygotes. The most frequent variant in *RAB27A* was c.560G>A (p.Arg187Gln), appearing in five alleles in five patients, including three single heterozygotes and two digenic/polygenic heterozygotes. Thirteen different variants in *AP3B1* were identified in 16 alleles in 16 patients, including 14 single heterozygotes, and two digenic/polygenic heterozygotes. There were no obvious repetitive same variants in *AP3B1* (Tables S1–S3).

For X‐linked genes, 10 different variants in *SH2D1A* were identified in 13 male patients and one female patient, respectively. Thirteen different variants in *XIAP* were identified in 13 male patients and two female patients, respectively. Three different variants in *MAGT1* were identified in three male patients. Variants c.158C>T (p.Thr53Ile) and c.163C>T (p.Arg55*) were the most frequent variants identified in *SH2D1A*, accounting for three alleles in three patients and three alleles in three patients, respectively (Tables S1–S3; Figure 4D–E).

Of 15 patients with digenic/polygenic heterozygous variants, *LYST* was the most frequently mutated gene and was identified in 11 patients, followed by *UNC13D* identified in nine patients. The most common concomitant mutation was *UNC13D*+*LYST*, which appeared in five patients. The other two common concomitant mutations were *UNC13D*+*RAB27A* and *LYST*+*STXBP2*, and both of them appeared in three patients (Table S3). In addition, three and one different variants in *ITK* and *CD27* were identified in three and one patients, respectively.

## 3 Discussion

In this study, 12 primary HLH‐associated genes were analyzed in 1224 patients with clinically confirmed or suspected HLH. To the best of our knowledge, this study has the largest patient sample size in China.

We found that 28.59% of the patients had at least one variant in the 12 genes. The frequency was lower than that reported by others, which varies from 30% to 80% among different ethnic groups [16, 17, 21, 22, 23, 24]. In addition, the median age of onset of our cohort was 24 years, older than that reported by others [15, 16, 17, 22]. There are two reasons for these differences. First, we included patients with both clinically confirmed and suspected HLH, while most studies included only clinically confirmed HLH patients [16, 18, 24]. Patients with clinically suspected HLH might have lower frequency of mutation. Second, 55.40% patients in our study were older than 18 years, while most studies mainly included pediatric patients [15, 16, 24]. The high proportion of adult patients can lead to the older median age of onset. Indeed, we found that primary HLH‐associated gene mutations happened less frequently in patients older than 18 years (24.61%) than in patients younger than 18 years (35.38%), which can also lead to the lower frequency of mutation.

As expected, a wider range of variants were found compared with previous studies conducted in Chinese patients due to larger sample size [17, 19]. A total of 275 different variants were found among 350 patients. Of these different mutations, most of them were missense mutations, and the proportion of frameshift variants and nonsense variants was low, which suggest that most of variants may partly impair rather than completely abrogate the function of affected proteins.

Another important finding was that most patients (64.86%) carried single heterozygous variants, while the proportion of patients with definitive genetic diagnosis was only 30.86%. This result is inconsistent with the study conducted in American, in which the proportion of patients with definitive genetic diagnosis (87%) was higher than that of patients with single heterozygous variants (13%) [15]. However, our result is consistent with the studies conducted in Chinese patients [17, 19]. This might reflect that primary HLH‐associated gene variants differ in different races.

In studies conducted in American [15], European [25], and Japanese [26] population, *PRF1* was reported as the most commonly mutated gene. However, the most frequently mutated gene in our study was *UNC13D*, which is consistent with studies conducted in both Chinese and Korean patients [17, 19, 27, 28]. The missense variant c.2588G>A (p.Gly863Asp) observed in 33 unrelated patients was the most common variant in *UNC13D* in our cohort. This variant was also the most common variant in two studies with Chinese patients but not in studies with patients of other ethnics [15, 16, 17, 19, 25, 29, 30]. Variant c.2588G>A (p.Gly863Asp) results in a non‐conservative amino acid change located in the MUN domain of *UNC13D*, which mediates localization on recycling endosomes and lysosomes [31]. The allele frequency of this variant in our cohort was higher than that in gnomAD population (39/2448 [1.59%] vs. 82/282,034 [0.03%], *χ* ^2^ = 1360, *p* < 0.001) and was categorized as pathogenic variant by ACMG and three prediction software. Variant c.1228A>C (p.Ile410Leu) was the second most common variant in *UNC13D*. The allele frequency of this variant in our cohort was also higher than that in gnomAD population (9/2448 [0.38%] vs. 327/282,242 [0.12%], *χ* ^2^ = 11, *p* < 0.001). All these data indicate the pathogenicity of these two variants. Consistent with our study, c.2588G>A (p.Gly863Asp) and c.1228A>C (p.Ile410Leu) were also the most common variants in one study conducted in Chinese patients [18]. Interestingly, the commonly reported variants in American (c.118‐308C>T), Italian (c.7531+1G>T) and Korean (c.754‐1G>C) patients were not found in our cohort [15, 16, 27], and only one patient carried variant c.1596+1G>C, which is common in Japanese patients [32, 33]. These data reflected the uniqueness of *UNC13D* mutation in China.

*PRF1* was the third most commonly mutated gene in our cohort. Compared with patients with other genes, the proportion of patients with definitive genetic diagnosis was highest in patients with *PRF1* mutation. The most common variant in *PRF1* was c.1349C>T (p.Thr450Met), which was only reported in Chinese patients [17, 29, 34]. The allele frequency of this variant in our cohort was higher than that in gnomAD population (21/2448 [0.86%] vs. 7/251,188 [0.002%], odds ratio = 310, 95%CI 127–878, *p* < 0.001) and was categorized as pathogenic variant by ACMG and three prediction software, which suggest its pathogenic role in HLH. The other two common variants, c.503G>A (p.Ser168Asn) and c.65del (p.Pro22fs), were also only reported in Chinese and Korean patients [17, 34, 35]. One study conducted in Chinese patients also found that the most common variant in *PRF1* was c.1349C>T (p.Thr450Met), followed by c.503G>A (p.Ser168Asn) and c.65del (p.Pro22fs) [29]. Interestingly, our study found five patients with concomitant variants in c.65del (p.Pro22fs) and c.503G>A (p.Ser168Asn). The commonly reported variants in American (c.50del (p.Leu17fs)), Italian (c.1122G>A (p. Trp374X)), and Japanese (c.1090_1091del (p.Leu364Glufs)) patients were not found in our cohort [15, 16, 33]. These data reflected the uniqueness of *PRF1* mutation in China. The frequency of another two FHL‐associated genes, *STXBP2* and *STX11*, was relatively low in our cohort. In addition, there were no patients with homozygous or compound heterozygous variants in *STX11* and only two patients carried compound heterozygous variants in *STXBP2*.

In our cohort, *LYST* was the second most commonly mutated gene. The high proportion of *LYST* mutation (22.00%) was also reported in studies conducted in Chinese patients, which were 14.58% (7/48) and 23.44% (30/128), respectively [18, 19]. Similarly to a previous study conducted in Chinese patients [19], variant c.368A>G (p.His123Arg) was also the most common variant of *LYST* in our cohort, which was rarely seen in other ethnic groups. The allele frequency of this variant in our cohort was higher than that in gnomAD population (6/2448 (0.11%) vs. 233/277,614 (0.08%), *χ* ^2^ = 5, *p* = 0.018). In addition, previous studies also identified this variant in six other HLH patients [18, 19, 36]. All these data suggest its pathogenic role in HLH. The other PID‐associated HLH genes, *RAB27A* and *AP3B1*, were also found in our cohort. However, most of patients with variants in *RAB27A* and *AP3B1* carried single heterozygous variants. There were no patients with homozygous or compound heterozygous variants in *AP3B1*, and only one patient carried homozygous variants and one patient carried compound heterozygous variants in *RAB27A*.

Variants in *SH2D1A* and *XIAP* were also common in our cohort, which do not impact lymphocyte cytotoxicity [37], but is characterized by extreme susceptibility to EBV [4, 13, 14]. Variants c.158C>T (p.Thr53Ile) and c.163C>T (p.Arg55*) were the two most common variants identified in *SH2D1A*. Both of them are classified as pathogenic by ACMG and have no information in population‐based database. Variant c.163C>T (p.Arg55*) was also reported in other studies [15, 18], while variant c.158C>T (p.Thr53Ile) was rarely described. The other primary HLH‐associated genes, *MAGT1*, *ITK*, and *CD27*, were rare in our cohort.

Fifteen patients with digenic/polygenic heterozygous variants were identified, which was common for HLH‐associated genes. The median age of onset of these patients was not significantly different from patients with definite genetic diagnosis, which suggests that digenic/polygenic heterozygous variants of different primary HLH‐associated genes may have a synergistic effect in the pathogenesis of HLH, as suggested by previous studies [38, 39].

This study had several limitations. First, t‐NGS‐based diagnostic approach is highly accurate in capturing single‐nucleotide variants, small deletions, and insertions variants; however, it cannot reliably identify large structural variations such as large deletions, duplications, or insertions. Second, some infrequent primary HLH‐associated genes such as *CTPS1*, *RASGRP1* [40], and *CDC42* [41] were not included in our panel, which could cause underdiagnosis. Third, lack of clinical information such as biochemical and immunologic data renders elucidation of the link between mutations and clinic‐pathological features of primary HLH impossible.

Overall, we described the largest mutation spectrum of primary HLH‐associated gene in Chinese HLH patients. Notably, missense mutation was the most common mutated type, and patients with single heterozygous variants were the predominant type of zygote. In addition, we found a small number of patients with digenic/polygenic heterozygous variants. Whether these gene mutations have a synergistic effect in the pathogenesis of HLH deserves further studies. More importantly, we found some characteristics of variants, which support racial specificity of primary HLH‐associated gene mutations. First, some common variants in our cohort, such as c.2588G>A (p.Gly863Asp) in *UNC13D*, c.368A>G (p.His123Arg) in *LYST*, and c.1349C>T (p.Thr450Met) in *PRF1*, were rarely found in other ethnic groups. Second, *UNC13D* was the most frequently mutated gene rather than *PRF1*. Third, *LYST* possessed relatively more variants; however, homozygous variants were not found. Lastly, some gene mutations, including *MAGT1*, *ITK*, and *CD27*, were rare in our cohort, which indicates that the t‐NGS‐based panel can exclude these genes to reduce the cost.

## 4 Materials and Methods

### 4.1 Patients and Samples

Medical records of patients with clinically confirmed HLH (meeting ≥ 5 HLH‐2004 diagnostic criteria [42]) or suspected HLH (meeting ≤ 4 criteria) were retrieved from the Fourth, Fifth, and Seventh Medical Centers of Chinese PLA General Hospital (all in Beijing, China), and Hainan Hospital of Chinese PLA General Hospital (Sanya, China) between January 2014 and September 2024. All patients underwent t‐NGS. Patients with biallelic variants or hemizygous mutations in primary HLH‐associated genes were defined as primary HLH patients. The study protocol was reviewed and approved by the Ethical Committee at the four hospitals. All procedures complied with the international ethical standards and the Helsinki Declaration.

### 4.2 Targeted Next‐Generation Sequencing

A t‐NGS panel covering 12 primary HLH‐associated genes (Table S4) was used. Genomic DNA was extracted from peripheral blood or bone marrow samples using the QIAsymphony DNA Mini Kit, and libraries were constructed using the QIAseq FX DNA Library Kit (both from Qiagen, Hilden, Germany). DNA sequencing was performed on an Illumina NovaSeq6000 system (Illumina, San Diego, CA, USA) with100 ng DNA input [3]. Sequence reads were aligned to the human reference genome (NCBI build 37/hg19).

### 4.3 Bioinformatics and Statistical Analyses

The Genome Aggregation Database (gnomAD; http://gnomad.broadinstitute.org/) was used to extract the frequencies of variants. Sequence variants were analyzed by Sorting Intolerant From Tolerant (SIFT), Polymorphism Phenotype v2 (Polyphen‐2), and Protein Variation Effect Analyzer (PROVEAN) [43, 44, 45]. Variants were categorized according to the American College of Medical Genetics and Genomics (ACMG) guidelines [46]. Only pathogenic, likely pathogenic, or uncertain significance variants were included. These variants also met at least one of the following requirements: (1) the mutation in the population has a minor allele frequency ≤ 0.01 based on the annotation from the gnomAD; (2) the mutation was determined to be harmful/possibly harmful by at least one of the three prediction algorithms (SIFT, Polyphen‐2, or PROVEAN); (3) the mutation had been reported to be pathogenic in the literature; or (4) the mutation was a nonsynonymous mutation that had not been previously reported in the literature and had no allele frequency annotation or pathogenicity prediction.

Non‐normally distributed data are presented as median and interquartile ranges (IQR), and categorical variables are presented as N. Two‐sample comparison of non‐normally distributed data is performed using Mann–Whitney *U* test. Fisher exact test or chi‐square test is used to compare categorical variables. All statistical analyses are performed by R Statistical Software (R Foundation for Statistical Computing). All *p* values are calculated as two‐sided, and *p* values less than 0.05 are considered statistically significant.

## Author Contributions

Conception and design: Wenshuai Zheng, Hongmei Ning, Xiaohong Li, and Lianming Liao. Acquisition and analysis of data: Wenshuai Zheng, Shenyu Wang, Zhenlan Du, and Xiaohong Li. Writing, review, and/or revision of the manuscript: Wenshuai Zheng, Shenyu Wang, Zhenlan Du, Hongmei Ning, Xiaohong Li, and Lianming Liao. Study supervision: Hongmei Ning, Xiaohong Li, and Lianming Liao. All authors read and approved the final version of the manuscript.

## Funding

The authors have nothing to report.

## Ethics Statement

The study protocol was approved by the institutional review board (IRB) of each participating center. The study was conducted according to the principles of the Declaration of Helsinki. The IRB approval number in the Fifth Medical Center of Chinese PLA General Hospital was KY‐2024‐9‐145‐1 (Data: 2024‐9‐30). The IRB approval number in Hainan Hospital of Chinese PLA General Hospital was S2024‐36‐02 (Data: 2024‐12‐25). The IRB approval number in the Fourth Medical Center of Chinese PLA General Hospital was 2024KY0125‐KS001 (Data: 2024‐12‐2). The IRB approval number in the Seventh Medical Center of Chinese PLA General Hospital was S2025‐020‐01 (Data: 2025‐3‐10). Since this study involved the analysis of existing data that were anonymized and de‐identified, the requirement for informed consent was waived by the IRB. All data were handled in compliance with relevant data protection regulations to ensure patient confidentiality.

## Conflicts of Interest

The authors declare no conflicts of interest.

## Supporting information




**Table S1**: Variants identified in 108 patients with a definitive genetic diagnosis.
**Table S2**: Variants identified in 227 patients with single heterozygous variants.
**Table S3**: Variants identified in 15 patients with digenic/polygenic variants.
**Table S4**: Twelve genes that are tested in targeted generation sequencing panel.

## Data Availability

All data are available from the corresponding authors upon request.
