# Supplementary material for: Spectrum of Primary Hemophagocytic Lymphohistiocytosis‐Associated Gene Mutations in Chinese Patients
Source: MedComm (2020). 2026 Sep 2;7(9):e70979. doi: 10.1002/mco2.70979 (PMC13535829; doi:10.1002/mco2.70979)
Supplement: Supplementary file 1 [file MCO2-7-e70979-s001.docx]

**Spectrum of primary hemophagocytic lymphohistiocytosis-associated gene mutations in Chinese patients**

Wenshuai Zheng^1*^, Shenyu Wang^2*^, Zhenlan Du^3*^, Lianming Liao^4＃^, Xiaohong Li^2＃^, Hongmei Ning^2＃^
^1^Department of Hematology, Hainan Hospital of Chinese PLA General Hospital, Sanya, Hainan 572000, China
^2^Senior Department of Hematology, Chinese PLA General Hospital, Beijing, China

^3^Senior Department of Pediatric, Chinese PLA General Hospital, Beijing, China

^4^Department of Laboratory Medicine, Fujian Medical University Union Hospital, Fuzhou, China

Corresponding Author: Dr. Hongmei Ning, Senior Department of Hematology, Chinese PLA General Hospital, Beijing, China, E-mail: ninghongmei72@sina.com
Dr. Xiaohong Li, Senior Department of Hematology, Chinese PLA General Hospital, Beijing, China, E-mail: lily.lxh@163.com

Dr. Lianming Liao, Department of Laboratory Medicine, Fujian Medical University Union Hospital, Fuzhou, China, E-mail: liaolianming@fjmu.edu.cn

^*^Wenshuai Zheng, Shenyu Wang and Zhenlan Du contributed equal to this study.
^＃^Hongmei Ning, Xiaohong Li and Lianming Liao are co-corresponding authors.

Table S1. Variants identified in 108 patients with a definitive genetic diagnosis.

| Patients | Age at testing, year | Sex | Gene | Reference sequence | Zygote | Variant | Type | Frequency in our chort, % | Frequency in gnomAD*, % | Polyphen2 | SIFT | PROVEAN | ACMG  classification |
| --- | --- | --- | --- | --- | --- | --- | --- | --- | --- | --- | --- | --- | --- |
| 1 | 41 | Male | UNC13D | NM_199242.3 | Hom | c.2588G>A (p.Gly863Asp) | Missense | 1.5931 | 0.02907 | probably damaging | Damaging | Deleterious | Possibly pathogenic |
| 2 | 1.7 | Female | UNC13D | NM_199242.2 | Hom | c.2588G>A (p.Gly863Asp) | Missense | 1.5931 | 0.02907 | probably damaging | Damaging | Deleterious | Possibly pathogenic |
| 3 | 63 | Male | UNC13D | NM_199242.3 | Hom | c.2588G>A (p.Gly863Asp) | Missense | 1.5931 | 0.02907 | probably damaging | Damaging | Deleterious | Possibly pathogenic |
| 4 | 30 | Female | UNC13D | NM_199242.2 | Hom | c.2588G>A (p.Gly863Asp) | Missense | 1.5931 | 0.02907 | probably damaging | Damaging | Deleterious | Possibly pathogenic |
| 5 | 18 | Male | UNC13D | NM_199242.2 | Hom | c.2588G>A (p.Gly863Asp) | Missense | 1.5931 | 0.02907 | probably damaging | Damaging | Deleterious | Possibly pathogenic |
| 6 | 2 | Male | UNC13D | NM_199242.2 | Hom | c.2588G>A (p.Gly863Asp) | Missense | 1.5931 | 0.02907 | probably damaging | Damaging | Deleterious | Possibly pathogenic |
| 7 | 0.2 | Male | UNC13D | NM_199242.3 | Hom | c.2709+1G>A | Intronic | 0.2042 | NA | NA | NA | NA | Possibly pathogenic |
| 8 | 2 | Male | UNC13D | NM_199242.2 | Hom | c.2759A>G (p.Tyr920Cys) | Missense | 0.0817 | NA | probably damaging | Damaging | Deleterious | Possibly pathogenic |
| 9 | 6 | Female | UNC13D | NM_199242.3 | Hom | c.3049G>A (p.Glu1017Lys) | Missense | 0.1225 | 0.0004351 | probably damaging | Damaging | Deleterious | Possibly pathogenic |
| 10 | 1 | Female | UNC13D | NM_199242.2 | Het | c.118-308C>T | Intronic | 0.0408 | NA | NA | NA | NA | Pathogenic |
|  |  |  | UNC13D | NM_199242.2 | Het | c.2190del (p.Val731fs) | Frameshift | 0.0408 | NA | NA | NA | NA | Possibly pathogenic |
| 11 | 11 | Male | UNC13D | NM_199242.3 | Het | c.518C>T (p.Thr173Met) | Missense | 0.0817 | 0.002798 | probably damaging | Damaging | Deleterious | Uncertain significance |
|  |  |  | UNC13D | NM_199242.3 | Het | c.706del (p.Asp236fs) | Frameshift | 0.0408 | NA | NA | NA | NA | Possibly pathogenic |
| 12 | 1 | Male | UNC13D | NM_199242.2 | Het | c.518C>T (p.Thr173Met) | Missense | 0.0817 | 0.002798 | probably damaging | Damaging | Deleterious | Uncertain significance |
|  |  |  | UNC13D | NM_199242.2 | Het | c.977C>T (p.Ser326Leu) | Missense | 0.0408 | 0.0007984 | benign | Damaging | Deleterious | Uncertain significance |
| 13 | 2.3 | Male | UNC13D | NM_199242.2 | Het | c.754-8C>T | Intronic | 0.0408 | 0.01176 | NA | NA | NA | Uncertain significance |
|  |  |  | UNC13D | NM_199242.2 | Het | c.3134C>T (p.Thr1045Met) | Missense | 0.1225 | 0.007472 | benign | Tolerated | Neutral | Uncertain significance |
| 14 | 0.1 | Male | UNC13D | NM_199242.2 | Het | c.755_756dup(p.Leu253fs) | Frameshift | 0.0817 | NA | NA | NA | NA | Pathogenic |
|  |  |  | UNC13D | NM_199242.2 | Het | c.1358del (p.Pro453fs) | Frameshift | 0.0817 | 0.0003993 | NA | NA | NA | Possibly pathogenic |
| 15 | 4 | Male | UNC13D | NM_199242.3 | Het | c.766C>T(p.Arg256*) | Nonsense | 0.0408 | 0.002488 | NA | NA | NA | Pathogenic |
|  |  |  | UNC13D | NM_199242.3 | Het | c.3049G>A (p.Glu1017Lys) | Missense | 0.1225 | 0.0004351 | probably damaging | Damaging | Deleterious | Possibly pathogenic |
| 16 | 25 | Female | UNC13D | NM_199242.2 | Het | c.817C>T (p.Arg273*) | Nonsense | 0.0817 | 0.002129 | NA | NA | NA | Pathogenic |
|  |  |  | UNC13D | NM_199242.2 | Het | c.1241G>T (p.Arg414Leu) | Missense | 0.0817 | 0.001418 | possibly damaging | Damaging | Deleterious | Possibly pathogenic |
| 17 | 14 | Female | UNC13D | NM_199242.3 | Het | c.1115T>C (p.Leu372Pro) | Missense | 0.0408 | 0.0007959 | probably damaging | Damaging | Deleterious | Uncertain significance |
|  |  |  | UNC13D | NM_199242.3 | Het | c.3053C>A (p.Ala1018Asp) | Missense | 0.0408 | 0.0008797 | probably damaging | Damaging | Deleterious | Possibly pathogenic |
| 18 | 3 | Male | UNC13D | NM_199242.2 | Het | c.1189G>A (p.Ala397Thr) | Missense | 0.0408 | 0.005587 | benign | Tolerated | Neutral | Uncertain significance |
|  |  |  | UNC13D | NM_199242.2 | Het | c.2257G>A (p.Gly753Ser) | Missense | 0.0408 | NA | benign | Tolerated | Neutral | Uncertain significance |
| 19 | 4 | Male | UNC13D | NM_199242.2 | Het | c.1228A>C (p.Ile410Leu) | Missense | 0.3676 | 0.1159 | benign | Tolerated | Neutral | Uncertain significance |
|  |  |  | UNC13D | NM_199242.2 | Het | c.2588G>A (p.Gly863Asp) | Missense | 1.5931 | 0.02907 | probably damaging | Damaging | Deleterious | Possibly pathogenic |
| 20 | 11 | Female | UNC13D | NM_199242.2 | Het | c.1228A>C (p.Ile410Leu) | Missense | 0.3676 | 0.1159 | benign | Tolerated | Neutral | Uncertain significance |
|  |  |  | UNC13D | NM_199242.2 | Het | c.2219C>T (p.Thr740Met) | Missense | 0.0408 | 0.01065 | probably damaging | Damaging | Neutral | Uncertain significance |
| 21 | 16 | Male | UNC13D | NM_199242.3 | Het | c.1358del (p.Pro453fs) | Frameshift | 0.0817 | 0.0003993 | NA | NA | NA | Possibly pathogenic |
|  |  |  | UNC13D | NM_199242.3 | Het | c.2588G>A (p.Gly863Asp) | Missense | 1.5931 | 0.02907 | probably damaging | Damaging | Deleterious | Possibly pathogenic |
| 22 | 31 | Male | UNC13D | NM_199242.3 | Het | c.1993-2A>G | Intronic | 0.0408 | NA | NA | NA | NA | Pathogenic |
|  |  |  | UNC13D | NM_199242.3 | Het | c.1241G>T (p.Arg414Leu) | Missense | 0.0817 | 0.001418 | possibly damaging | Damaging | Deleterious | Possibly pathogenic |
| 23 | 3 | Male | UNC13D | NM_199242.2 | Het | c.2038C>G (p.Arg680Gly) | Missense | 0.0408 | NA | probably damaging | Damaging | Deleterious | Uncertain significance |
|  |  |  | UNC13D | NM_199242.2 | Het | c.2368-1G>T | Intronic | 0.0408 | NA | NA | NA | NA | Pathogenic |
| 24 | 31 | Male | UNC13D | NM_199242.3 | Het | c.2164G>T (p.Glu722*) | Nonsense | 0.0408 | NA | NA | NA | NA | Possibly pathogenic |
|  |  |  | UNC13D | NM_199242.3 | Het | c.2588G>A (p.Gly863Asp) | Missense | 1.5931 | 0.02907 | probably damaging | Damaging | Deleterious | Possibly pathogenic |
| 25 | 3 | Female | UNC13D | NM_199242.3 | Het | c.2240G>A (p.Ser747Asn) | Missense | 0.0817 | 0.003595 | benign | Tolerated | Neutral | Uncertain significance |
|  |  |  | UNC13D | NM_199242.3 | Het | c.2955-4G>A | Intronic | 0.0408 | NA | NA | NA | NA | Uncertain significance |
| 26 | 9.5 | Female | UNC13D | NM_199242.2 | Het | c.2240G>A (p.Ser747Asn) | Missense | 0.0817 | 0.003595 | benign | Tolerated | Neutral | Uncertain significance |
|  |  |  | UNC13D | NM_199242.2 | Het | c.3229C>T (p.Arg1077Trp) | Missense | 0.0408 | 0.002347 | probably damaging | Damaging | Deleterious | Uncertain significance |
| 27 | 39 | Male | UNC13D | NM_199242.3 | Het | c.2588G>A (p.Gly863Asp) | Missense | 1.5931 | 0.02907 | probably damaging | Damaging | Deleterious | Possibly pathogenic |
|  |  |  | UNC13D | NM_199242.3 | Het | c.3193C>T (p.Arg1065*) | Nonsense | 0.0817 | 0.00107 | NA | NA | NA | Pathogenic |
| 28 | 0.2 | Female | UNC13D | NM_199242.2 | Het | c.2709+1G>A | Intronic | 0.2042 | NA | NA | NA | NA | Possibly pathogenic |
|  |  |  | UNC13D | NM_199242.2 | Het | c.3193C>T (p.Arg1065*) | Nonsense | 0.0817 | 0.00107 | NA | NA | NA | Pathogenic |
| 29 | 2.3 | Female | UNC13D | NM_199242.3 | Het | c.2955-5C>T | Intronic | 0.0408 | 0.001543 | NA | NA | NA | Uncertain significance |
|  |  |  | UNC13D | NM_199242.3 | Het | c.2955-12C>G | Intronic | 0.0408 | NA | NA | NA | NA | Uncertain significance |
| 30 | 10.5 | Female | UNC13D | NM_199242.2 | Het | c.3037G>A (p.Asp1013Asn) | Missense | 0.0408 | 0.005276 | probably damaging | Damaging | Deleterious | Uncertain significance |
|  |  |  | UNC13D | NM_199242.2 | Het | c.3134C>T (p.Thr1045Met) | Missense | 0.1225 | 0.007472 | benign | Tolerated | Neutral | Uncertain significance |
| 31 | 12 | Female | PRF1 | NM_001083116.2 | Hom | c.46C>T (p.Pro16Ser) | Missense | 0.1225 | 0.0004356 | possibly damaging | Tolerated | Neutral | Uncertain significance |
| 32 | 10.6 | Male | PRF1 | NM_001083116.3 | Hom | c.394G>A (p.Gly132Arg) | Missense | 0.1225 | 0.000398 | possibly damaging | Damaging | Deleterious | Possibly pathogenic |
| 33 | 11 | Female | PRF1 | NM_001083116.2 | Hom | c.673C>T (p.Arg225Trp) | Missense | 0.1634 | 0.001202 | possibly damaging | Damaging | Deleterious | Pathogenic |
| 34 | 34 | Female | PRF1 | NM_001083116.2 | Hom | c.1066C>T (p.Arg356Trp) | Missense | 0.1634 | 0.001429 | benign | Damaging | Deleterious | Pathogenic |
| 35 | 9 | Male | PRF1 | NM_001083116.3 | Hom | c.1066C>T (p.Arg356Trp) | Missense | 0.1634 | 0.001429 | benign | Damaging | Deleterious | Pathogenic |
| 36 | 10 | Male | PRF1 | NM_001083116.1 | Hom | c.1349C>T (p.Thr450Met) | Missense | 0.8578 | 0.002787 | possibly damaging | Damaging | Deleterious | Pathogenic |
| 37 | 17 | Female | PRF1 | NM_001083116.3 | Hom | c.1349C>T (p.Thr450Met) | Missense | 0.8578 | 0.002787 | possibly damaging | Damaging | Deleterious | Pathogenic |
| 38 | 7 | Male | PRF1 | NM_001083116.2 | Hom | c.1349C>T (p.Thr450Met) | Missense | 0.8578 | 0.002787 | possibly damaging | Damaging | Deleterious | Pathogenic |
| 39 | 17 | Male | PRF1 | NM_001083116.1 | Hom | c.1349C>T (p.Thr450Met) | Missense | 0.8578 | 0.002787 | possibly damaging | Damaging | Deleterious | Pathogenic |
| 40 | 9.2 | Female | PRF1 | NM_001083116.2 | Hom | c.1349C>T (p.Thr450Met) | Missense | 0.8578 | 0.002787 | possibly damaging | Damaging | Deleterious | Pathogenic |
| 41 | 17 | Male | PRF1 | NM_001083116.2 | Hom | c.1349C>T (p.Thr450Met) | Missense | 0.8578 | 0.002787 | possibly damaging | Damaging | Deleterious | Pathogenic |
| 42 | 10.9 | Female | PRF1 | NM_001083116.2 | Hom | c.1349C>T (p.Thr450Met) | Missense | 0.8578 | 0.002787 | possibly damaging | Damaging | Deleterious | Pathogenic |
| 43 | 9 | Male | PRF1 | NM_001083116.3 | Hom | c.1349C>T (p.Thr450Met) | Missense | 0.8578 | 0.002787 | possibly damaging | Damaging | Deleterious | Pathogenic |
| 44 | 55 | Male | PRF1 | NM_001083116.3 | Het | c.46C>T (p.Pro16Ser) | Missense | 0.1225 | 0.0004356 | possibly damaging | Tolerated | Neutral | Uncertain significance |
|  |  |  | PRF1 | NM_001083116.3 | Het | c.503G>A (p.Ser168Asn) | Missense | 0.3676 | 0.003537 | benign | Tolerated | Neutral | Possibly pathogenic |
| 45 | 0.1 | Male | PRF1 | NM_001083116.3 | Het | c.65del (p.Pro22fs) | Frameshift | 0.2859 | 0.00125 | NA | NA | NA | Pathogenic |
|  |  |  | PRF1 | NM_001083116.3 | Het | c.503G>A (p.Ser168Asn) | Missense | 0.3676 | 0.003537 | benign | Tolerated | Neutral | Possibly pathogenic |
| 46 | 28 | Male | PRF1 | NM_001083116.1 | Het | c.65del (p.Pro22fs) | Frameshift | 0.2859 | 0.00125 | NA | NA | NA | Pathogenic |
|  |  |  | PRF1 | NM_001083116.3 | Het | c.503G>A (p.Ser168Asn) | Missense | 0.3676 | 0.003537 | benign | Tolerated | Neutral | Possibly pathogenic |
| 47 | 58 | Male | PRF1 | NM_001083116.3 | Het | c.65del (p.Pro22fs) | Frameshift | 0.2859 | 0.00125 | NA | NA | NA | Pathogenic |
|  |  |  | PRF1 | NM_001083116.3 | Het | c.503G>A (p.Ser168Asn) | Missense | 0.3676 | 0.003537 | benign | Tolerated | Neutral | Possibly pathogenic |
| 48 | 59 | Male | PRF1 | NM_001083116.3 | Het | c.65del (p.Pro22fs) | Frameshift | 0.2859 | 0.00125 | NA | NA | NA | Pathogenic |
|  |  |  | PRF1 | NM_001083116.3 | Het | c.503G>A (p.Ser168Asn) | Missense | 0.3676 | 0.003537 | benign | Tolerated | Neutral | Possibly pathogenic |
| 49 | 0.1 | Female | PRF1 | NM_001083116.3 | Het | c.65del (p.Pro22fs) | Frameshift | 0.2859 | 0.00125 | NA | NA | NA | Pathogenic |
|  |  |  | PRF1 | NM_001083116.3 | Het | c.503G>A (p.Ser168Asn) | Missense | 0.3676 | 0.003537 | benign | Tolerated | Neutral | Possibly pathogenic |
| 50 | 0.1 | Male | PRF1 | NM_001083116.1 | Het | c.65del (p.Pro22fs) | Frameshift | 0.2859 | 0.00125 | NA | NA | NA | Pathogenic |
|  |  |  | PRF1 | NM_001083116.2 | Het | c.1213del (p.Gln405fs) | Frameshift | 0.0408 | NA | NA | NA | NA | Pathogenic |
| 51 | 34 | Male | PRF1 | NM_001083116.2 | Het | c.65del (p.Pro22fs) | Frameshift | 0.2859 | 0.00125 | NA | NA | NA | Pathogenic |
|  |  |  | PRF1 | NM_001083116.2 | Het | c.182T>C (p.Val61Ala) | Missense | 0.0817 | 0.003595 | probably damaging | Damaging | Deleterious | Uncertain significance |
| 52 | 3 | Male | PRF1 | NM_001083116.1 | Het | c.122C>T (p.Ala41Val) | Missense | 0.0408 | NA | benign | Tolerated | Neutral | Uncertain significance |
|  |  |  | PRF1 | NM_001083116.1 | Het | c.1427G>T (p.Gly476Val) | Missense | 0.0408 | 0.001193 | possibly damaging | Damaging | Deleterious | Uncertain significance |
| 53 | 27 | Male | PRF1 | NM_001083116.3 | Het | c.139G>T（p.Gly47Cys） | Missense | 0.0408 | 0.003185 | probably damaging | Damaging | Deleterious | Uncertain significance |
|  |  |  | PRF1 | NM_001083116.3 | Het | c.503G>A (p.Ser168Asn) | Missense | 0.3676 | 0.003537 | benign | Tolerated | Neutral | Possibly pathogenic |
| 54 | 11 | Male | PRF1 | NM_001083116.2 | Het | c.147C>A (p.Asp49Glu) | Missense | 0.0817 | 0.0004035 | probably damaging | Damaging | Deleterious | Uncertain significance |
|  |  |  | PRF1 | NM_001083116.2 | Het | c.1349C>T (p.Thr450Met) | Missense | 0.8578 | 0.002787 | possibly damaging | Damaging | Deleterious | Pathogenic |
| 55 | 26 | Male | PRF1 | NM_001083116.3 | Het | c.182T>C (p.Val61Ala) | Missense | 0.0817 | 0.003595 | probably damaging | Damaging | Deleterious | Uncertain significance |
|  |  |  | PRF1 | NM_001083116.3 | Het | c.1168C>T (p.Arg390*) | Nonsense | 0.0408 | 0.0004051 | NA | NA | NA | Possibly pathogenic |
| 56 | 1 | Male | PRF1 | NM_001083116.1 | Het | c.377G>A (p.Arg126His) | Missense | 0.0408 | 0.0016 | benign | Tolerated | Neutral | Uncertain significance |
|  |  |  | PRF1 | NM_001083116.1 | Het | c.1153C>T (p.Arg385Trp) | Missense | 0.0817 | 0.01695 | benign | Damaging | Neutral | Uncertain significance |
| 57 | 12 | Female | PRF1 | NM_001083116.3 | Het | c.394G>A (p.Gly132Arg) | Missense | 0.1225 | 0.000398 | possibly damaging | Damaging | Deleterious | Possibly pathogenic |
|  |  |  | PRF1 | NM_001083116.3 | Het | c.673C>T (p.Arg225Trp) | Missense | 0.1634 | 0.001202 | possibly damaging | Damaging | Deleterious | Pathogenic |
| 58 | 40 | Male | PRF1 | NM_001083116.3 | Het | c.445G>A (p.Gly149Ser) | Missense | 0.0408 | 0.01379 | probably damaging | Damaging | Deleterious | Pathogenic |
|  |  |  | PRF1 | NM_001083116.3 | Het | c.1153C>T (p.Arg385Trp) | Missense | 0.0817 | 0.01695 | benign | Damaging | Neutral | Uncertain significance |
| 59 | 8 | Female | PRF1 | NM_001083116.3 | Het | c.527G>A (p.Cys176Tyr) | Missense | 0.0408 | NA | probably damaging | Damaging | Deleterious | Uncertain significance |
|  |  |  | PRF1 | NM_001083116.3 | Het | c.1422del (p.Leu478fs) | Frameshift | 0.0408 | 0.0003977 | NA | NA | NA | Possibly pathogenic |
| 60 | 1.9 | Female | PRF1 | NM_001083116.2 | Het | c.784G>A (p.Ala262Thr) | Missense | 0.0408 | NA | possibly damaging | Damaging | Deleterious | Uncertain significance |
|  |  |  | PRF1 | NM_001083116.2 | Het | c.983G>C (p.Trp328Ser) | Missense | 0.0408 | NA | probably damaging | Damaging | Deleterious | Uncertain significance |
| 61 | 2 | Male | LYST | NM_000081.4 | Het | c.52C>T (p.Arg18Trp) | Missense | 0.0408 | 0.001597 | possibly damaging | Damaging | Deleterious | Uncertain significance |
|  |  |  | LYST | NM_000081.4 | Het | c.4589G>C (p.Arg1530Thr) | Missense | 0.0408 | 0.06539 | benign | Tolerated | Neutral | Uncertain significance |
| 62 | 29 | Male | LYST | NM_000081.3 | Het | c.6796G>C(p.Val2266Leu) | Missense | 0.0408 | 0.001197 | benign | Damaging | Neutral | Uncertain significance |
|  |  |  | LYST | NM_000081.3 | Het | c.6079G>C (p.Val2027Leu) | Missense | 0.0817 | 0.006027 | benign | Damaging | Neutral | Uncertain significance |
| 63 | 7 | Female | LYST | NM_000081.3 | Het | c.368A>G (p.His123Arg) | Missense | 0.2451 | 0.08393 | benign | Tolerated | Neutral | Uncertain significance |
|  |  |  | LYST | NM_000081.3 | Het | c.6775G>A (p.Val2259Ile) | Missense | 0.1225 | 0.006383 | benign | Tolerated | Neutral | Uncertain significance |
| 64 | 29 | Female | LYST | NM_000081.3 | Het | c.439C>T (p.Arg147Cys) | Missense | 0.0408 | 0.0003993 | probably damaging | Damaging | Deleterious | Uncertain significance |
|  |  |  | LYST | NM_000081.3 | Het | c.7748A>G (p.His2583Arg) | Missense | 0.0408 | 0.001194 | benign | Tolerated | Deleterious | Uncertain significance |
| 65 | 22 | Male | LYST | NM_000081.3 | Het | c.452A>G (p.His151Arg) | Missense | 0.1225 | 0.0124 | benign | Damaging | Deleterious | Uncertain significance |
|  |  |  | LYST | NM_000081.4 | Het | c.3362A>G (p.Gln1121Arg) | Missense | 0.1225 | 0.001195 | benign | Tolerated | Neutral | Uncertain significance |
| 66 | 1.5 | Female | LYST | NM_000081.4 | Het | c.830A>T (p.His277Leu) | Missense | 0.1225 | 0.0007978 | benign | Tolerated | Deleterious | Uncertain significance |
|  |  |  | LYST | NM_000081.4 | Het | c.5213T>C (p.Ile1738Thr) | Missense | 0.0408 | 0.0004668 | possibly damaging | Tolerated | Neutral | Uncertain significance |
| 67 | 74 | Male | LYST | NM_000081.4 | Het | c.1183C>T (p.Arg395Cys) | Missense | 0.0817 | 0.002479 | benign | Tolerated | Deleterious | Uncertain significance |
|  |  |  | LYST | NM_000081.4 | Het | c.2813G>T (p.Ser938Ile) | Missense | 0.0817 | 0.002126 | benign | Tolerated | Neutral | Uncertain significance |
| 68 | 33 | Female | LYST | NM_000081.4 | Het | c.1183C>T (p.Arg395Cys) | Missense | 0.0817 | 0.002479 | benign | Tolerated | Deleterious | Uncertain significance |
|  |  |  | LYST | NM_000081.4 | Het | c.2813G>T (p.Ser938Ile) | Missense | 0.0817 | 0.002126 | benign | Tolerated | Neutral | Uncertain significance |
| 69 | 28 | Female | LYST | NM_000081.4 | Het | c.3362A>G (p.Gln1121Arg) | Missense | 0.1225 | 0.001195 | benign | Tolerated | Neutral | Uncertain significance |
|  |  |  | LYST | NM_000081.4 | Het | c.4863-11T>G | Intronic | 0.0408 | 0.02846 | NA | NA | NA | Uncertain significance |
| 70 | 69 | Male | LYST | NM_000081.3 | Het | c.3841G>A (p.Ala1281Thr) | Missense | 0.0408 | NA | benign | Tolerated | Neutral | Uncertain significance |
|  |  |  | LYST | NM_000081.3 | Het | c.7910C>T (p.Thr2637Met) | Missense | 0.0408 | 0.00557 | possibly damaging | Damaging | Neutral | Uncertain significance |
| 71 | 31 | Male | LYST | NM_000081.3 | Het | c.4275del (p.Leu1425fs) | Frameshift | 0.0408 | NA | NA | NA | NA | Possibly pathogenic |
|  |  |  | LYST | NM_000081.3 | Het | c.6775G>A (p.Val2259Ile) | Missense | 0.1225 | 0.006383 | benign | Tolerated | Neutral | Uncertain significance |
| 72 | 57 | Male | LYST | NM_000081.4 | Het | c.4689-9T>C | Intronic | 0.0408 | NA | NA | NA | NA | Uncertain significance |
|  |  |  | LYST | NM_000081.4 | Het | c.7661A>G (p.Gln2554Arg) | Missense | 0.0817 | 0.00199 | benign | Tolerated | Neutral | Uncertain significance |
| 73 | 2 | Male | LYST | NM_000081.4 | Het | c.6158T>C (p.Met2053Thr) | Missense | 0.0408 | NA | benign | Tolerated | Neutral | Uncertain significance |
|  |  |  | LYST | NM_000081.4 | Het | c.8514C>A (p.Asp2838Glu) | Missense | 0.0817 | 0.002388 | benign | Tolerated | Neutral | Uncertain significance |
| 74 | 21 | Female | LYST | NM_000081.3 | Het | c.8624G>A (p.Arg2875His) | Missense | 0.0408 | 0.02267 | probably damaging | Damaging | Deleterious | Uncertain significance |
|  |  |  | LYST | NM_000081.3 | Het | c.8975T>C (p.Leu2992Pro) | Missense | 0.0408 | NA | probably damaging | Damaging | Deleterious | Uncertain significance |
| 75 | 66 | Male | SH2D1A | NM_002351.4 | Hemi | c.7G>T (p.Ala3Ser) | Missense | 0.0408 | 0.01363 | benign | Tolerated | Neutral | Uncertain significance |
| 76 | 1.7 | Male | SH2D1A | NM_002351.4 | Hemi | c.21T>G (p.Tyr7*) | Nonsense | 0.0408 | NA | NA | NA | NA | Pathogenic |
| 77 | 0.8 | Male | SH2D1A | NM_002351.4 | Hemi | c.137T>C (p.Leu46Pro) | Missense | 0.0408 | NA | probably damaging | Damaging | Deleterious | Uncertain significance |
| 78 | 11 | Male | SH2D1A | NM_002351.4 | Hemi | c.158C>T (p.Thr53Ile) | Missense | 0.1225 | NA | probably damaging | Damaging | Deleterious | Pathogenic |
| 79 | 7 | Male | SH2D1A | NM_002351.4 | Hemi | c.158C>T (p.Thr53Ile) | Missense | 0.1225 | NA | probably damaging | Damaging | Deleterious | Pathogenic |
| 80 | 3 | Male | SH2D1A | NM_002351.4 | Hemi | c.158C>T (p.Thr53Ile) | Missense | 0.1225 | NA | probably damaging | Damaging | Deleterious | Pathogenic |
| 81 | 1 | Male | SH2D1A | NM_002351.4 | Hemi | c.163C>T (p.Arg55*) | Nonsense | 0.1225 | NA | NA | NA | NA | Pathogenic |
| 82 | 2 | Male | SH2D1A | NM_002351.4 | Hemi | c.163C>T (p.Arg55*) | Nonsense | 0.1225 | NA | NA | NA | NA | Pathogenic |
| 83 | 2 | Male | SH2D1A | NM_002351.4 | Hemi | c.163C>T (p.Arg55*) | Nonsense | 0.1225 | NA | NA | NA | NA | Pathogenic |
| 84 | 8 | Male | SH2D1A | NM_002351.4 | Hemi | c.169T>C (p.Ser57Pro) | Missense | 0.0408 | NA | possibly damaging | Tolerated | Neutral | Uncertain significance |
| 85 | 0.9 | Male | SH2D1A | NM_002351.4 | Hemi | c.201+2T>C | Intronic | 0.0408 | NA | NA | NA | NA | Possibly pathogenic |
| 86 | 13 | Male | SH2D1A | NM_002351.5 | Hemi | c.203C>T (p.Thr68Ile) | Missense | 0.0408 | NA | possibly damaging | Damaging | Deleterious | Possibly pathogenic |
| 87 | 66 | Male | SH2D1A | NM_002351.4 | Hemi | c.287T>A (p.Ile96Lys) | Missense | 0.0408 | NA | benign | Tolerated | Neutral | Uncertain significance |
| 88 | 5 | Male | XIAP | NM_001167.3 | Hemi | c.500T>A (p.Leu167*) | Nonsense | 0.0408 | NA | NA | NA | NA | Pathogenic |
| 89 | 0.3 | Male | XIAP | NM_001167.4 | Hemi | c.587A>T (p.Asp196Val) | Missense | 0.0408 | NA | probably damaging | Damaging | Deleterious | Uncertain significance |
| 90 | 10 | Male | XIAP | NM_001167.3 | Hemi | c.592G>A (p.Val198Met) | Missense | 0.0408 | NA | probably damaging | Damaging | Deleterious | Uncertain significance |
| 91 | 0.8 | Male | XIAP | NM_001167.4 | Hemi | c.612_614del (p.Gly205del) | InFrame | 0.0817 | NA | NA | NA | NA | Possibly pathogenic |
| 92 | 2 | Male | XIAP | NM_001167.4 | Hemi | c.612_614del (p.Gly205del) | InFrame | 0.0817 | NA | NA | NA | NA | Possibly pathogenic |
| 93 | 5 | Male | XIAP | NM_001167.3 | Hemi | c.664C>T (p.Arg222*) | Nonsense | 0.0408 | NA | NA | NA | NA | Pathogenic |
| 94 | 21 | Male | XIAP | NM_001167.4 | Hemi | c.712C>T (p.Arg238*) | Nonsense | 0.0408 | NA | NA | NA | NA | Pathogenic |
| 95 | 3 | Male | XIAP | NM_001167.3 | Hemi | c.723_724del (p.Asp242fs) | Frameshift | 0.0408 | NA | NA | NA | NA | Pathogenic |
| 96 | 38 | Male | XIAP | NM_001167.3 | Hemi | c.878-4G>A | Intronic | 0.0408 | 0.00218 | NA | NA | NA | Uncertain significance |
| 97 | 15 | Male | XIAP | NM_001167.4 | Hemi | c.894_898del (p.Lys299fs) | Frameshift | 0.0817 | NA | NA | NA | NA | Pathogenic |
| 98 | 13 | Male | XIAP | NM_001167.3 | Hemi | c.894_898del (p.Lys299fs) | Frameshift | 0.0817 | NA | NA | NA | NA | Pathogenic |
| 99 | 8 | Male | XIAP | NM_001167.3 | Hemi | c.1021_1022del (p.Asn341fs) | Frameshift | 0.0408 | NA | NA | NA | NA | Pathogenic |
| 100 | 6.2 | Male | XIAP | NM_001167.4 | Hemi | c.1315_1315dup (p.Glu439fs) | Frameshift | 0.0408 | NA | NA | NA | NA | Uncertain significance |
| 101 | 58 | Male | MAGT1 | NM_032121.5 | Hemi | c.95_95dup (p.Asn32fs) | Frameshift | 0.0408 | NA | NA | NA | NA | Uncertain significance |
| 102 | 7 | Male | MAGT1 | NM_032121.5 | Hemi | c.565C>T (p.Gln189*) | Nonsense | 0.0408 | NA | NA | NA | NA | Uncertain significance |
| 103 | 28 | Male | MAGT1 | NM_032121.5 | Hemi | c.713G>A (p.Arg238Gln) | Missense | 0.0408 | 0.001768 | NA | Damaging | Deleterious | Possibly pathogenic |
| 104 | 0.2 | Female | STXBP2 | NM_006949.4 | Het | c.560C>T (p.Pro187Leu) | Missense | 0.0408 | 0.0006397 | probably damaging | Damaging | Deleterious | Uncertain significance |
|  |  |  | STXBP2 | NM_006949.4 | Het | c.985_986del (p.Ile329fs) | Frameshift | 0.0408 | NA | NA | NA | NA | Possibly pathogenic |
| 105 | 0.7 | Male | STXBP2 | NM_006949.4 | Het | c.1058G>T (p.Cys353Phe) | Missense | 0.0408 | NA | probably damaging | Damaging | Deleterious | Uncertain significance |
|  |  |  | STXBP2 | NM_006949.4 | Het | c.1214G>A (p.Arg405Gln) | Missense | 0.0817 | 0.001597 | probably damaging | Damaging | Deleterious | Possibly pathogenic |
| 106 | 13 | Female | RAB27A | NM_004580.5 | Hom | c.239+3A>G | Intronic | 0.0817 | NA | NA | NA | NA | Uncertain significance |
| 107 | 30 | Female | RAB27A | NM_004580.5 | Het | c.305C>T (p.Thr102Ile) | Missense | 0.0408 | NA | probably damaging | Damaging | Deleterious | Uncertain significance |
|  |  |  | RAB27A | NM_004580.5 | Het | c.598C>T (p.Arg200*) | Nonsense | 0.0408 | 0.0003976 | NA | NA | NA | Possibly pathogenic |
| 108 | 2 | Female | ITK | NM_005546.4 | Hom | c.1060+1G>T | Intronic | 0.0817 | NA | NA | NA | NA | Possibly pathogenic |

*gnomAD v2.1.1 population frequency. ACMG, American College of Medical Genetics and Genomics; Het, heterozygous; Hom, homozygous; Hemi, hemizygous; NA, not available; Polyphen-2, Polymorphism Phenotype v2; PROVEAN, Protein Variation Effect Analyzer; SIFT, Sorting Intolerant From Tolerant.

Table S2. Variants identified in 227 patients with single heterozygous variants.

| Patients | Age at testing, year | Sex | Gene | Reference sequence | Zygosity | Variant | Type | Frequency in our chort, % | Frequency in gnomAD*, % | Polyphen2 | SIFT | PROVEAN | ACMG  classification |
| --- | --- | --- | --- | --- | --- | --- | --- | --- | --- | --- | --- | --- | --- |
| 1 | 1 | Male | UNC13D | NM_199242.2 | Het | c.46C>T (p.Arg16Cys) | Missense | 0.0408 | 0.00201 | probably damaging | Damaging | Neutral | Uncertain significance |
| 2 | 64 | Male | UNC13D | NM_199242.2 | Het | c.68G>A (p.Arg23His) | Missense | 0.0408 | 0.006413 | benign | Damaging | Neutral | Uncertain significance |
| 3 | 35 | Male | UNC13D | NM_199242.3 | Het | c.205C>T (p.His69Tyr) | Missense | 0.0817 | 0.002006 | benign | Tolerated | Neutral | Uncertain significance |
| 4 | 61 | Male | UNC13D | NM_199242.3 | Het | c.205C>T (p.His69Tyr) | Missense | 0.0817 | 0.002006 | benign | Tolerated | Neutral | Uncertain significance |
| 5 | 3 | Male | UNC13D | NM_199242.3 | Het | c.569+5G>A | Intronic | 0.0408 | 0.0004031 | NA | NA | NA | Uncertain significance |
| 6 | 44 | Male | UNC13D | NM_199242.2 | Het | c.602A>G (p.His201Arg) | Missense | 0.2042 | 0.006718 | possibly damaging | Damaging | Deleterious | Uncertain significance |
| 7 | NA | Male | UNC13D | NM_199242.2 | Het | c.602A>G (p.His201Arg) | Missense | 0.2042 | 0.006718 | possibly damaging | Damaging | Deleterious | Uncertain significance |
| 8 | 20 | Female | UNC13D | NM_199242.2 | Het | c.602A>G (p.His201Arg) | Missense | 0.2042 | 0.006718 | possibly damaging | Damaging | Deleterious | Uncertain significance |
| 9 | 36 | Male | UNC13D | NM_199242.2 | Het | c.602A>G (p.His201Arg) | Missense | 0.2042 | 0.006718 | possibly damaging | Damaging | Deleterious | Uncertain significance |
| 10 | 3.2 | Female | UNC13D | NM_199242.2 | Het | c.602A>G (p.His201Arg) | Missense | 0.2042 | 0.006718 | possibly damaging | Damaging | Deleterious | Uncertain significance |
| 11 | 29 | Male | UNC13D | NM_199242.3 | Het | c.680G>A (p.Arg227His) | Missense | 0.0817 | 0.00566 | possibly damaging | Tolerated | Neutral | Uncertain significance |
| 12 | 2 | Male | UNC13D | NM_199242.3 | Het | c.680G>A (p.Arg227His) | Missense | 0.0817 | 0.00566 | possibly damaging | Tolerated | Neutral | Uncertain significance |
| 13 | 2 | Male | UNC13D | NM_199242.2 | Het | c.755_756dup (p.Leu253fs) | Frameshift | 0.0817 | NA | NA | NA | NA | Pathogenic |
| 14 | 27 | Male | UNC13D | NM_199242.2 | Het | c.760C>T (p.Arg254Cys) | Missense | 0.0408 | 0.01992 | benign | Damaging | Neutral | Uncertain significance |
| 15 | 55 | Male | UNC13D | NM_199242.2 | Het | c.796C>T (p.Arg266Cys) | Missense | 0.0408 | 0.002486 | benign | Tolerated | Deleterious | Uncertain significance |
| 16 | 23 | Female | UNC13D | NM_199242.3 | Het | c.817C>T (p.Arg273*) | Nonsense | 0.0817 | 0.002129 | NA | NA | NA | Pathogenic |
| 17 | 2.1 | Male | UNC13D | NM_199242.2 | Het | c.856C>T (p.Arg286Trp) | Missense | 0.0408 | 0.00568 | possibly damaging | Damaging | Deleterious | Uncertain significance |
| 18 | 2 | Male | UNC13D | NM_199242.2 | Het | c.859-3C>T | Intronic | 0.0817 | 0.003961 | NA | NA | NA | Uncertain significance |
| 19 | 52 | Female | UNC13D | NM_199242.2 | Het | c.859-3C>T | Intronic | 0.0817 | 0.003961 | NA | NA | NA | Uncertain significance |
| 20 | 67 | Female | UNC13D | NM_199242.2 | Het | c.878G>A (p.Arg293His) | Missense | 0.0408 | 0.0004039 | possibly damaging | Damaging | Neutral | Uncertain significance |
| 21 | 2 | Female | UNC13D | NM_199242.3 | Het | c.929C>T (p.Ser310Phe) | Missense | 0.0408 | 0.0004004 | possibly damaging | Damaging | Deleterious | Uncertain significance |
| 22 | 47 | Female | UNC13D | NM_199242.2 | Het | c.1034C>G (p.Ser345Cys) | Missense | 0.0817 | NA | probably damaging | Damaging | Deleterious | Uncertain significance |
| 23 | 53 | Male | UNC13D | NM_199242.2 | Het | c.1034C>G (p.Ser345Cys) | Missense | 0.0817 | NA | probably damaging | Damaging | Deleterious | Uncertain significance |
| 24 | 0.2 | Male | UNC13D | NM_199242.2 | Het | c.1056-1G>C | Intronic | 0.0408 | NA | NA | NA | NA | Pathogenic |
| 25 | 23 | Female | UNC13D | NM_199242.2 | Het | c.1201_1202delinsAA (p.Ser401Asn) | Missense | 0.0408 | NA | benign | Tolerated | Neutral | Uncertain significance |
| 26 | 45 | Male | UNC13D | NM_199242.2 | Het | c.1228A>C (p.Ile410Leu) | Missense | 0.3676 | 0.1159 | benign | Tolerated | Neutral | Uncertain significance |
| 27 | 66 | Male | UNC13D | NM_199242.2 | Het | c.1228A>C (p.Ile410Leu) | Missense | 0.3676 | 0.1159 | benign | Tolerated | Neutral | Uncertain significance |
| 28 | 23 | Female | UNC13D | NM_199242.2 | Het | c.1228A>C (p.Ile410Leu) | Missense | 0.3676 | 0.1159 | benign | Tolerated | Neutral | Uncertain significance |
| 29 | 26 | Male | UNC13D | NM_199242.2 | Het | c.1228A>C (p.Ile410Leu) | Missense | 0.3676 | 0.1159 | benign | Tolerated | Neutral | Uncertain significance |
| 30 | 10 | Female | UNC13D | NM_199242.2 | Het | c.1232G>A (p.Arg411Gln) | Missense | 0.0408 | 0.05953 | benign | Tolerated | Neutral | Uncertain significance |
| 31 | 3 | Male | UNC13D | NM_199242.3 | Het | c.1279C>T (p.Arg427Trp) | Missense | 0.0408 | 0.002835 | probably damaging | Damaging | Deleterious | Uncertain significance |
| 32 | 5 | Female | UNC13D | NM_199242.3 | Het | c.1280G>A (p.Arg427Gln) | Missense | 0.0408 | 0.007443 | possibly damaging | Damaging | Deleterious | Uncertain significance |
| 33 | 13 | Female | UNC13D | NM_199242.2 | Het | c.1407G>C (p.Trp469Cys) | Missense | 0.0408 | NA | probably damaging | Damaging | Deleterious | Uncertain significance |
| 34 | 33 | Female | UNC13D | NM_199242.2 | Het | c.1596+1G>C | Intronic | 0.0408 | NA | NA | NA | NA | Pathogenic |
| 35 | 60 | Female | UNC13D | NM_199242.3 | Het | c.1783T>C (p.Ser595Pro) | Missense | 0.0408 | NA | benign | Tolerated | Neutral | Uncertain significance |
| 36 | 20 | Male | UNC13D | NM_199242.3 | Het | c.1992G>C (p.Glu664Asp) | Missense | 0.0408 | 0.0003978 | benign | Tolerated | Neutral | Uncertain significance |
| 37 | 38 | Female | UNC13D | NM_199242.3 | Het | c.2243C>T (p.Ala748Val) | Missense | 0.1634 | 0.009188 | benign | Tolerated | Neutral | Uncertain significance |
| 38 | 24 | Male | UNC13D | NM_199242.3 | Het | c.2243C>T (p.Ala748Val) | Missense | 0.1634 | 0.009188 | benign | Tolerated | Neutral | Uncertain significance |
| 39 | NA | Female | UNC13D | NM_199242.2 | Het | c.2243C>T (p.Ala748Val) | Missense | 0.1634 | 0.009188 | benign | Tolerated | Neutral | Uncertain significance |
| 40 | 3.5 | Male | UNC13D | NM_199242.2 | Het | c.2243C>T (p.Ala748Val) | Missense | 0.1634 | 0.009188 | benign | Tolerated | Neutral | Uncertain significance |
| 41 | 31 | Male | UNC13D | NM_199242.2 | Het | c.2488G>A (p.Val830Met) | Missense | 0.0408 | NA | benign | Tolerated | Neutral | Uncertain significance |
| 42 | 16 | Male | UNC13D | NM_199242.2 | Het | c.2495C>T (p.Ala832Val) | Missense | 0.0408 | 0.003206 | benign | Tolerated | Neutral | Uncertain significance |
| 43 | 30 | Male | UNC13D | NM_199242.2 | Het | c.2588G>A (p.Gly863Asp) | Missense | 1.5931 | 0.02907 | probably damaging | Damaging | Deleterious | Possibly pathogenic |
| 44 | 0.8 | Male | UNC13D | NM_199242.2 | Het | c.2588G>A (p.Gly863Asp) | Missense | 1.5931 | 0.02907 | probably damaging | Damaging | Deleterious | Possibly pathogenic |
| 45 | 24 | Male | UNC13D | NM_199242.2 | Het | c.2588G>A (p.Gly863Asp) | Missense | 1.5931 | 0.02907 | probably damaging | Damaging | Deleterious | Possibly pathogenic |
| 46 | 4 | Male | UNC13D | NM_199242.2 | Het | c.2588G>A (p.Gly863Asp) | Missense | 1.5931 | 0.02907 | probably damaging | Damaging | Deleterious | Possibly pathogenic |
| 47 | 2.1 | Female | UNC13D | NM_199242.2 | Het | c.2588G>A (p.Gly863Asp) | Missense | 1.5931 | 0.02907 | probably damaging | Damaging | Deleterious | Possibly pathogenic |
| 48 | 40 | Female | UNC13D | NM_199242.2 | Het | c.2588G>A (p.Gly863Asp) | Missense | 1.5931 | 0.02907 | probably damaging | Damaging | Deleterious | Possibly pathogenic |
| 49 | 25 | Female | UNC13D | NM_199242.2 | Het | c.2588G>A (p.Gly863Asp) | Missense | 1.5931 | 0.02907 | probably damaging | Damaging | Deleterious | Possibly pathogenic |
| 50 | 7 | Female | UNC13D | NM_199242.2 | Het | c.2588G>A (p.Gly863Asp) | Missense | 1.5931 | 0.02907 | probably damaging | Damaging | Deleterious | Possibly pathogenic |
| 51 | 28 | Male | UNC13D | NM_199242.2 | Het | c.2588G>A (p.Gly863Asp) | Missense | 1.5931 | 0.02907 | probably damaging | Damaging | Deleterious | Possibly pathogenic |
| 52 | 29 | Male | UNC13D | NM_199242.2 | Het | c.2588G>A (p.Gly863Asp) | Missense | 1.5931 | 0.02907 | probably damaging | Damaging | Deleterious | Possibly pathogenic |
| 53 | 5 | Female | UNC13D | NM_199242.2 | Het | c.2588G>A (p.Gly863Asp) | Missense | 1.5931 | 0.02907 | probably damaging | Damaging | Deleterious | Possibly pathogenic |
| 54 | 8.1 | Female | UNC13D | NM_199242.2 | Het | c.2588G>A (p.Gly863Asp) | Missense | 1.5931 | 0.02907 | probably damaging | Damaging | Deleterious | Possibly pathogenic |
| 55 | 66 | Female | UNC13D | NM_199242.2 | Het | c.2588G>A (p.Gly863Asp) | Missense | 1.5931 | 0.02907 | probably damaging | Damaging | Deleterious | Possibly pathogenic |
| 56 | 55 | Male | UNC13D | NM_199242.2 | Het | c.2588G>A (p.Gly863Asp) | Missense | 1.5931 | 0.02907 | probably damaging | Damaging | Deleterious | Possibly pathogenic |
| 57 | 24 | Female | UNC13D | NM_199242.2 | Het | c.2588G>A (p.Gly863Asp) | Missense | 1.5931 | 0.02907 | probably damaging | Damaging | Deleterious | Possibly pathogenic |
| 58 | 7.1 | Female | UNC13D | NM_199242.2 | Het | c.2588G>A (p.Gly863Asp) | Missense | 1.5931 | 0.02907 | probably damaging | Damaging | Deleterious | Possibly pathogenic |
| 59 | 38 | Female | UNC13D | NM_199242.2 | Het | c.2588G>A (p.Gly863Asp) | Missense | 1.5931 | 0.02907 | probably damaging | Damaging | Deleterious | Possibly pathogenic |
| 60 | 24 | Male | UNC13D | NM_199242.2 | Het | c.2588G>A (p.Gly863Asp) | Missense | 1.5931 | 0.02907 | probably damaging | Damaging | Deleterious | Possibly pathogenic |
| 61 | 54 | Male | UNC13D | NM_199242.2 | Het | c.2588G>A (p.Gly863Asp) | Missense | 1.5931 | 0.02907 | probably damaging | Damaging | Deleterious | Possibly pathogenic |
| 62 | 26 | Female | UNC13D | NM_199242.2 | Het | c.2588G>A (p.Gly863Asp) | Missense | 1.5931 | 0.02907 | probably damaging | Damaging | Deleterious | Possibly pathogenic |
| 63 | 34 | Female | UNC13D | NM_199242.2 | Het | c.2588G>A (p.Gly863Asp) | Missense | 1.5931 | 0.02907 | probably damaging | Damaging | Deleterious | Possibly pathogenic |
| 64 | 29 | Female | UNC13D | NM_199242.2 | Het | c.2588G>A (p.Gly863Asp) | Missense | 1.5931 | 0.02907 | probably damaging | Damaging | Deleterious | Possibly pathogenic |
| 65 | 24 | Male | UNC13D | NM_199242.2 | Het | c.2594C>A (p.Pro865Gln) | Missense | 0.0408 | NA | probably damaging | Damaging | Deleterious | Uncertain significance |
| 66 | 2 | Male | UNC13D | NM_199242.2 | Het | c.2709+1G>A | Intronic | 0.2042 | NA | NA | NA | NA | Possibly pathogenic |
| 67 | 31 | Male | UNC13D | NM_199242.2 | Het | c.2711C>T (p.Ala904Val) | Missense | 0.0408 | NA | benign | Tolerated | Neutral | Uncertain significance |
| 68 | 76 | Male | UNC13D | NM_199242.2 | Het | c.271G>A (p.Val91Met) | Missense | 0.0408 | 0.001812 | benign | Tolerated | Neutral | Uncertain significance |
| 69 | 43 | Female | UNC13D | NM_199242.3 | Het | c.2756C>T (p.Ser919Phe) | Missense | 0.1225 | 0.004278 | possibly damaging | Tolerated | Neutral | Uncertain significance |
| 70 | 54 | Male | UNC13D | NM_199242.2 | Het | c.2756C>T (p.Ser919Phe) | Missense | 0.1225 | 0.004278 | possibly damaging | Tolerated | Neutral | Uncertain significance |
| 71 | 3.2 | Male | UNC13D | NM_199242.2 | Het | c.2800G>A (p.Ala934Thr) | Missense | 0.0408 | 0.0004792 | probably damaging | Damaging | Deleterious | Uncertain significance |
| 72 | 61 | Female | UNC13D | NM_199242.2 | Het | c.2828A>G (p.Asn943Ser) | Missense | 0.0408 | 0.0663 | possibly damaging | Damaging | Deleterious | Uncertain significance |
| 73 | 68 | Female | UNC13D | NM_199242.3 | Het | c.2972C>T (p.Pro991Leu) | Missense | 0.0408 | 0.002581 | benign | Tolerated | Neutral | Uncertain significance |
| 74 | 6 | Female | UNC13D | NM_199242.3 | Het | c.2986G>A (p.Gly996Arg) | Missense | 0.0408 | NA | probably damaging | Damaging | Deleterious | Uncertain significance |
| 75 | 7 | Male | UNC13D | NM_199242.2 | Het | c.3134C>T (p.Thr1045Met) | Missense | 0.1225 | 0.007472 | benign | Tolerated | Neutral | Uncertain significance |
| 76 | 40 | Male | UNC13D | NM_199242.3 | Het | c.3140C>T (p.Pro1047Leu) | Missense | 0.0817 | 0.002012 | probably damaging | Damaging | Deleterious | Uncertain significance |
| 77 | 26 | Male | UNC13D | NM_199242.3 | Het | c.3140C>T (p.Pro1047Leu) | Missense | 0.0817 | 0.002012 | probably damaging | Damaging | Deleterious | Uncertain significance |
| 78 | 9 | Female | UNC13D | NM_199242.2 | Het | c.3229_3235del (p.Arg1077fs) | Frameshift | 0.0408 | NA | NA | NA | NA | Uncertain significance |
| 79 | 47 | Male | UNC13D | NM_199242.2 | Het | c.3232G>C (p.Ala1078Pro) | Missense | 0.0408 | NA | possibly damaging | Tolerated | Neutral | Uncertain significance |
| 80 | 4.2 | Female | LYST | NM_000081.3 | Het | c.235C>G (p.Leu79Val) | Missense | 0.0408 | NA | probably damaging | Damaging | Neutral | Uncertain significance |
| 81 | 28 | Male | LYST | NM_000081.4 | Het | c.262G>A (p.Val88Ile) | Missense | 0.0408 | NA | probably damaging | Damaging | Neutral | Uncertain significance |
| 82 | 1 | Female | LYST | NM_000081.3 | Het | c.368A>G (p.His123Arg) | Missense | 0.2451 | 0.08393 | benign | Tolerated | Neutral | Uncertain significance |
| 83 | 4 | Female | LYST | NM_000081.3 | Het | c.368A>G (p.His123Arg) | Missense | 0.2451 | 0.08393 | benign | Tolerated | Neutral | Uncertain significance |
| 84 | 4 | Male | LYST | NM_000081.3 | Het | c.368A>G (p.His123Arg) | Missense | 0.2451 | 0.08393 | benign | Tolerated | Neutral | Uncertain significance |
| 85 | 26 | Female | LYST | NM_000081.3 | Het | c.368A>G (p.His123Arg) | Missense | 0.2451 | 0.08393 | benign | Tolerated | Neutral | Uncertain significance |
| 86 | 17 | Male | LYST | NM_000081.3 | Het | c.731T>C (p.Leu244Ser) | Missense | 0.0408 | NA | benign | Damaging | Neutral | Uncertain significance |
| 87 | 18 | Male | LYST | NM_000081.4 | Het | c.830A>T (p.His277Leu) | Missense | 0.1225 | 0.0007978 | benign | Tolerated | Deleterious | Uncertain significance |
| 88 | 41 | Female | LYST | NM_000081.4 | Het | c.830A>T (p.His277Leu) | Missense | 0.1225 | 0.0007978 | benign | Tolerated | Deleterious | Uncertain significance |
| 89 | 0.2 | Male | LYST | NM_000081.3 | Het | c.857T>G (p.Val286Gly) | Missense | 0.0408 | NA | probably damaging | Damaging | Deleterious | Uncertain significance |
| 90 | 27 | Female | LYST | NM_000081.4 | Het | c.956C>T (p.Pro319Leu) | Missense | 0.0408 | 0.001198 | probably damaging | Tolerated | Deleterious | Uncertain significance |
| 91 | 75 | Female | LYST | NM_000081.3 | Het | c.1348C>T (p.Leu450Phe) | Missense | 0.0408 | NA | probably damaging | Tolerated | Neutral | Uncertain significance |
| 92 | 54 | Male | LYST | NM_000081.4 | Het | c.1481C>T (p.Ser494Leu) | Missense | 0.0408 | 0.003898 | probably damaging | Damaging | Deleterious | Uncertain significance |
| 93 | 49 | Female | LYST | NM_000081.3 | Het | c.1555C>G (p.Leu519Val) | Missense | 0.0408 | NA | probably damaging | Damaging | Neutral | Uncertain significance |
| 94 | 68 | Male | LYST | NM_000081.3 | Het | c.2172A>G (p.Ile724Met) | Missense | 0.0408 | 0.0071 | benign | Tolerated | Neutral | Uncertain significance |
| 95 | 0.4 | Female | LYST | NM_000081.4 | Het | c.2474T>C (p.Ile825Thr) | Missense | 0.0408 | 0.001194 | benign | Tolerated | Neutral | Uncertain significance |
| 96 | 7 | Male | LYST | NM_000081.4 | Het | c.3352A>G (p.Arg1118Gly) | Missense | 0.0408 | NA | benign | Tolerated | Deleterious | Uncertain significance |
| 97 | NA | Female | LYST | NM_000081.4 | Het | c.3362A>G (p.Gln1121Arg) | Missense | 0.1225 | 0.001195 | benign | Tolerated | Neutral | Uncertain significance |
| 98 | 21 | Male | LYST | NM_000081.3 | Het | c.3466C>G (p.Leu1156Val) | Missense | 0.0408 | NA | probably damaging | Tolerated | Neutral | Uncertain significance |
| 99 | 25 | Female | LYST | NM_000081.3 | Het | c.3611C>G (p.Ala1204Gly) | Missense | 0.0408 | NA | benign | Damaging | Neutral | Uncertain significance |
| 100 | 39 | Male | LYST | NM_000081.4 | Het | c.4573A>G (p.Ile1525Val) | Missense | 0.0817 | 0.001418 | benign | Tolerated | Neutral | Uncertain significance |
| 101 | 1.2 | Male | LYST | NM_000081.4 | Het | c.4573A>G (p.Ile1525Val) | Missense | 0.0817 | 0.001418 | benign | Tolerated | Neutral | Uncertain significance |
| 102 | 2 | Male | LYST | NM_000081.4 | Het | c.4592T>C (p.Leu1531Pro) | Missense | 0.0408 | NA | benign | Tolerated | Neutral | Uncertain significance |
| 103 | NA | Male | LYST | NM_000081.4 | Het | c.4952G>C (p.Cys1651Ser) | Missense | 0.0408 | 0.002391 | benign | Damaging | Deleterious | Uncertain significance |
| 104 | 13 | Male | LYST | NM_000081.3 | Het | c.5290G>A (p.Gly1764Ser) | Missense | 0.0408 | 0.00239 | benign | Tolerated | Neutral | Uncertain significance |
| 105 | 30 | Male | LYST | NM_000081.4 | Het | c.5435G>A (p.Gly1812Asp) | Missense | 0.0408 | 0.001063 | probably damaging | Tolerated | Deleterious | Uncertain significance |
| 106 | 3 | Male | LYST | NM_000081.4 | Het | c.5519C>T (p.Ser1840Leu) | Missense | 0.0408 | 0.0003995 | benign | Tolerated | Deleterious | Uncertain significance |
| 107 | 51 | Female | LYST | NM_000081.4 | Het | c.5663A>T (p.Asp1888Val) | Missense | 0.0408 | 0.0003989 | benign | Tolerated | Deleterious | Uncertain significance |
| 108 | 24 | Male | LYST | NM_000081.4 | Het | c.5689G>A (p.Glu1897Lys) | Missense | 0.0408 | NA | benign | Tolerated | Neutral | Uncertain significance |
| 109 | 65 | Female | LYST | NM_000081.4 | Het | c.6418_6429del (p.Val2140_Gln2143del) | InFrame | 0.0408 | NA | NA | NA | NA | Uncertain significance |
| 110 | 55 | Male | LYST | NM_000081.3 | Het | c.6735C>G (p.Ser2245Arg) | Missense | 0.0817 | NA | possibly damaging | Damaging | Neutral | Uncertain significance |
| 111 | 40 | Male | LYST | NM_000081.4 | Het | c.6767C>T (p.Ser2256Phe) | Missense | 0.1225 | NA | benign | Damaging | Neutral | Uncertain significance |
| 112 | 10 | Female | LYST | NM_000081.4 | Het | c.6767C>T (p.Ser2256Phe) | Missense | 0.1225 | NA | benign | Damaging | Neutral | Uncertain significance |
| 113 | 37 | Male | LYST | NM_000081.4 | Het | c.6767C>T (p.Ser2256Phe) | Missense | 0.1225 | NA | benign | Damaging | Neutral | Uncertain significance |
| 114 | 2.9 | Male | LYST | NM_000081.3 | Het | c.6874G>A (p.Ala2292Thr) | Missense | 0.0408 | 0.0007984 | benign | Tolerated | Neutral | Uncertain significance |
| 115 | 38 | Male | LYST | NM_000081.3 | Het | c.7392G>A (p.Met2464Ile) | Missense | 0.0408 | 0.0003985 | benign | Tolerated | Neutral | Uncertain significance |
| 116 | 1.2 | Male | LYST | NM_000081.4 | Het | c.7400A>G (p.Asp2467Gly) | Missense | 0.0817 | 0.001594 | probably damaging | Damaging | Deleterious | Uncertain significance |
| 117 | 2 | Male | LYST | NM_000081.4 | Het | c.7400A>G (p.Asp2467Gly) | Missense | 0.0817 | 0.001594 | probably damaging | Damaging | Deleterious | Uncertain significance |
| 118 | 70 | Male | LYST | NM_000081.4 | Het | c.7493G>A (p.Cys2498Tyr) | Missense | 0.0817 | 0.0003992 | probably damaging | Tolerated | Deleterious | Uncertain significance |
| 119 | 6 | Female | LYST | NM_000081.3 | Het | c.7493G>A (p.Cys2498Tyr) | Missense | 0.0817 | 0.0003992 | probably damaging | Tolerated | Deleterious | Uncertain significance |
| 120 | 6 | Female | LYST | NM_000081.4 | Het | c.7604A>T (p.Asn2535Ile) | Missense | 0.0408 | NA | benign | Tolerated | Neutral | Uncertain significance |
| 121 | 1 | Female | LYST | NM_000081.4 | Het | c.7661A>G (p.Gln2554Arg) | Missense | 0.0817 | 0.00199 | benign | Tolerated | Neutral | Uncertain significance |
| 122 | 75 | Female | LYST | NM_000081.4 | Het | c.7766G>A (p.Arg2589Gln) | Missense | 0.0408 | 0.0007963 | benign | Tolerated | Neutral | Uncertain significance |
| 123 | 15 | Male | LYST | NM_000081.4 | Het | c.7832G>A (p.Arg2611His) | Missense | 0.0408 | 0.004776 | probably damaging | Damaging | Neutral | Uncertain significance |
| 124 | 29 | Female | LYST | NM_000081.4 | Het | c.7915C>T (p.Leu2639Phe) | Missense | 0.0408 | NA | benign | Tolerated | Neutral | Uncertain significance |
| 125 | 10.6 | Male | LYST | NM_000081.4 | Het | c.7919C>T (p.Ala2640Val) | Missense | 0.0817 | 0.00358 | benign | Tolerated | Neutral | Uncertain significance |
| 126 | 67 | Male | LYST | NM_000081.4 | Het | c.7919C>T (p.Ala2640Val) | Missense | 0.0817 | 0.00358 | benign | Tolerated | Neutral | Uncertain significance |
| 127 | 51 | Male | LYST | NM_000081.4 | Het | c.8050G>A (p.Glu2684Lys) | Missense | 0.0408 | 0.02469 | benign | Damaging | Neutral | Uncertain significance |
| 128 | 51 | Male | LYST | NM_000081.4 | Het | c.8273C>T (p.Ala2758Val) | Missense | 0.0408 | NA | benign | Tolerated | Neutral | Uncertain significance |
| 129 | 22 | Female | LYST | NM_000081.3 | Het | c.8351C>G (p.Ser2784Cys) | Missense | 0.0817 | 0.001196 | benign | Damaging | Deleterious | Uncertain significance |
| 130 | 12 | Male | LYST | NM_000081.4 | Het | c.8480A>G (p.Lys2827Arg) | Missense | 0.0408 | NA | benign | Tolerated | Neutral | Uncertain significance |
| 131 | 6.6 | Female | LYST | NM_000081.3 | Het | c.8514C>A (p.Asp2838Glu) | Missense | 0.0817 | 0.002388 | benign | Tolerated | Neutral | Uncertain significance |
| 132 | 0.3 | Female | LYST | NM_000081.4 | Het | c.8833A>G (p.Thr2945Ala) | Missense | 0.0408 | 0.001591 | benign | Tolerated | Neutral | Uncertain significance |
| 133 | 32 | Female | LYST | NM_000081.3 | Het | c.9514G>A (p.Asp3172Asn) | Missense | 0.0408 | 0.001415 | probably damaging | Damaging | Deleterious | Uncertain significance |
| 134 | 1.2 | Male | LYST | NM_000081.4 | Het | c.9958C>T (p.Arg3320Trp) | Missense | 0.0817 | 0.001595 | probably damaging | Damaging | Deleterious | Uncertain significance |
| 135 | 35 | Female | LYST | NM_000081.4 | Het | c.9958C>T (p.Arg3320Trp) | Missense | 0.0817 | 0.001595 | probably damaging | Damaging | Deleterious | Uncertain significance |
| 136 | 68 | Male | LYST | NM_000081.4 | Het | c.10234C>T (p.Arg3412Cys) | Missense | 0.0408 | 0.002476 | probably damaging | Damaging | Deleterious | Uncertain significance |
| 137 | 56 | Male | LYST | NM_000081.3 | Het | c.10317G>T (p.Leu3439Phe) | Missense | 0.0408 | 0.001193 | benign | Tolerated | Neutral | Uncertain significance |
| 138 | 32 | Female | LYST | NM_000081.3 | Het | c.10658G>A (p.Ser3553Asn) | Missense | 0.0408 | 0.0003976 | benign | Tolerated | Neutral | Uncertain significance |
| 139 | 34 | Female | LYST | NM_000081.4 | Het | c.10744A>G (p.Thr3582Ala) | Missense | 0.0408 | 0.001591 | possibly damaging | Tolerated | Neutral | Uncertain significance |
| 140 | 50 | Male | LYST | NM_000081.3 | Het | c.10851A>T (p.Glu3617Asp) | Missense | 0.0817 | NA | benign | Tolerated | Neutral | Uncertain significance |
| 141 | 18 | Female | LYST | NM_000081.4 | Het | c.10851A>T (p.Glu3617Asp) | Missense | 0.0817 | NA | benign | Tolerated | Neutral | Uncertain significance |
| 142 | NA | Male | LYST | NM_000081.4 | Het | c.10941G>C (p.Arg3647Ser) | Missense | 0.0408 | 0.002392 | probably damaging | Tolerated | Neutral | Uncertain significance |
| 143 | 3.2 | Female | PRF1 | NM_001083116.1 | Het | c.10C>T (p.Arg4Cys) | Missense | 0.0408 | 0.1093 | benign | Damaging | Neutral | Uncertain significance |
| 144 | 11 | Female | PRF1 | NM_001083116.1 | Het | c.82C>T (p.Arg28Cys) | Missense | 0.0408 | 0.1103 | probably damaging | Damaging | Neutral | Pathogenic |
| 145 | 10 | Female | PRF1 | NM_001083116.3 | Het | c.147C>A (p.Asp49Glu) | Missense | 0.0817 | 0.0004035 | probably damaging | Damaging | Deleterious | Uncertain significance |
| 146 | 4 | Female | PRF1 | NM_001083116.3 | Het | c.148G>C (p.Val50Leu) | Missense | 0.0408 | 0.004844 | benign | Damaging | Neutral | Uncertain significance |
| 147 | 2 | Female | PRF1 | NM_001083116.2 | Het | c.164G>A (p.Arg55His) | Missense | 0.0408 | 0.007846 | benign | Tolerated | Deleterious | Uncertain significance |
| 148 | 4 | Male | PRF1 | NM_001083116.2 | Het | c.208G>A (p.Asp70Asn) | Missense | 0.0408 | 0.003196 | benign | Tolerated | Neutral | Uncertain significance |
| 149 | 1 | Female | PRF1 | NM_001083116.2 | Het | c.272C>T (p.Ala91Val) | Missense | 0.0408 | 2.916 | possibly damaging | Damaging | Deleterious | Uncertain significance |
| 150 | 51 | Male | PRF1 | NM_001083116.2 | Het | c.286C>T (p.Arg96Trp) | Missense | 0.0408 | 0.002811 | probably damaging | Damaging | Deleterious | Uncertain significance |
| 151 | 1.5 | Male | PRF1 | NM_001083116.2 | Het | c.503G>A (p.Ser168Asn) | Missense | 0.3676 | 0.003537 | benign | Tolerated | Neutral | Possibly pathogenic |
| 152 | 1.8 | Male | PRF1 | NM_001083116.3 | Het | c.503G>A (p.Ser168Asn) | Missense | 0.3676 | 0.003537 | benign | Tolerated | Neutral | Possibly pathogenic |
| 153 | 8 | Female | PRF1 | NM_001083116.3 | Het | c.673C>T (p.Arg225Trp) | Missense | 0.1634 | 0.001202 | possibly damaging | Damaging | Deleterious | Pathogenic |
| 154 | 1 | Female | PRF1 | NM_001083116.2 | Het | c.674G>A (p.Arg225Gln) | Missense | 0.0408 | 0.01387 | benign | Tolerated | Neutral | Uncertain significance |
| 155 | 52 | Male | PRF1 | NM_001083116.3 | Het | c.832G>A (p.Ala278Thr) | Missense | 0.0408 | NA | benign | Tolerated | Neutral | Pathogenic |
| 156 | 4 | Female | PRF1 | NM_001083116.3 | Het | c.889C>T (p.Arg297Trp) | Missense | 0.0817 | 0.0007954 | possibly damaging | Damaging | Deleterious | Uncertain significance |
| 157 | 1.7 | Male | PRF1 | NM_001083116. 2 | Het | c.889C>T (p.Arg297Trp) | Missense | 0.0817 | 0.0007954 | possibly damaging | Damaging | Deleterious | Uncertain significance |
| 158 | 15 | Male | PRF1 | NM_001083116.2 | Het | c.1033C>T (p.Pro345Ser) | Missense | 0.0408 | 0.006385 | probably damaging | Tolerated | Deleterious | Uncertain significance |
| 159 | 30 | Female | PRF1 | NM_001083116. 2 | Het | c.1213C>T (p.Gln405*) | Nonsense | 0.0408 | 0.0004023 | NA | NA | NA | Pathogenic |
| 160 | 43 | Male | PRF1 | NM_001083116.1 | Het | c.1228C>T (p.Arg410Trp) | Missense | 0.0408 | 0.005001 | possibly damaging | Damaging | Deleterious | Pathogenic |
| 161 | 69 | Female | PRF1 | NM_001083116.2 | Het | c.1244C>G (p.Ala415Gly) | Missense | 0.0408 | 0.000803 | benign | Tolerated | Deleterious | Uncertain significance |
| 162 | 1.2 | Female | PRF1 | NM_001083116.3 | Het | c.1266C>G (p.Ile422Met) | Missense | 0.0408 | NA | possibly damaging | Tolerated | Neutral | Uncertain significance |
| 163 | 17 | Male | PRF1 | NM_001083116.2 | Het | c.1312T>C (p.Tyr438His) | Missense | 0.0408 | 0.0003987 | probably damaging | Damaging | Deleterious | Uncertain significance |
| 164 | 5.2 | Male | PRF1 | NM_001083116.3 | Het | c.1349C>T (p.Thr450Met) | Missense | 0.8578 | 0.002787 | possibly damaging | Damaging | Deleterious | Pathogenic |
| 165 | 35 | Female | PRF1 | NM_001083116.1 | Het | c.1349C>T (p.Thr450Met) | Missense | 0.8578 | 0.002787 | possibly damaging | Damaging | Deleterious | Pathogenic |
| 166 | 1.4 | Female | PRF1 | NM_001083116.3 | Het | c.1349C>T (p.Thr450Met) | Missense | 0.8578 | 0.002787 | possibly damaging | Damaging | Deleterious | Pathogenic |
| 167 | 29 | Female | PRF1 | NM_001083116.1 | Het | c.1349C>T (p.Thr450Met) | Missense | 0.8578 | 0.002787 | possibly damaging | Damaging | Deleterious | Pathogenic |
| 168 | 51 | Female | PRF1 | NM_001083116.2 | Het | c.1391G>A (p.Arg464Gln) | Missense | 0.0408 | 0.002475 | benign | Tolerated | Neutral | Uncertain significance |
| 169 | 57 | Male | PRF1 | NM_001083116.2 | Het | c.1427_1428delins (p.Gly476fs) | Frameshift | 0.0408 | NA | NA | NA | NA | Possibly pathogenic |
| 170 | NA | Female | PRF1 | NM_001083116.3 | Het | c.1616C>T (p.Pro539Leu) | Missense | 0.0408 | NA | possibly damaging | Damaging | Deleterious | Uncertain significance |
| 171 | 55 | Female | STXBP2 | NM_006949.4 | Het | c.109A>G (p.Ser37Gly) | Missense | 0.0408 | NA | benign | Tolerated | Neutral | Uncertain significance |
| 172 | 11 | Female | STXBP2 | NM_006949.3 | Het | c.184A>G (p.Asn62Asp) | Missense | 0.0408 | 0.002787 | possibly damaging | Tolerated | Neutral | Uncertain significance |
| 173 | 3 | Female | STXBP2 | NM_006949.4 | Het | c.400C>G (p.His134Asp) | Missense | 0.0408 | NA | benign | Tolerated | Neutral | Uncertain significance |
| 174 | 3.3 | Male | STXBP2 | NM_006949.3 | Het | c.493C>T (p.Arg165Cys) | Missense | 0.0408 | NA | probably damaging | Damaging | Deleterious | Uncertain significance |
| 175 | 0.8 | Male | STXBP2 | NM_006949.4 | Het | c.530C>T (p.Ala177Val) | Missense | 0.0408 | NA | probably damaging | Tolerated | Deleterious | Uncertain significance |
| 176 | 1.8 | Male | STXBP2 | NM_006949.4 | Het | c.569G>A (p.Arg190His) | Missense | 0.0408 | NA | probably damaging | Damaging | Deleterious | Uncertain significance |
| 177 | 4 | Male | STXBP2 | NM_006949.4 | Het | c.575G>A (p.Arg192His) | Missense | 0.0817 | 0.003807 | probably damaging | Damaging | Deleterious | Uncertain significance |
| 178 | 0.8 | Female | STXBP2 | NM_006949.3 | Het | c.575G>A (p.Arg192His) | Missense | 0.0817 | 0.003807 | probably damaging | Damaging | Deleterious | Uncertain significance |
| 179 | 1.1 | Female | STXBP2 | NM_006949.4 | Het | c.646A>G (p.Thr216Ala) | Missense | 0.0408 | 0.001417 | benign | Tolerated | Neutral | Uncertain significance |
| 180 | 2.2 | Female | STXBP2 | NM_006949.4 | Het | c.761A>G (p.Tyr254Cys) | Missense | 0.0408 | NA | probably damaging | Damaging | Deleterious | Uncertain significance |
| 181 | 1.6 | Male | STXBP2 | NM_006949.4 | Het | c.939C>A (p.Ser313Arg) | Missense | 0.0408 | NA | possibly damaging | Damaging | Deleterious | Uncertain significance |
| 182 | 3.7 | Female | STXBP2 | NM_006949.3 | Het | c.953C>T (p.Thr318Met) | Missense | 0.0408 | 0.03894 | probably damaging | Tolerated | Neutral | Uncertain significance |
| 183 | 46 | Female | STXBP2 | NM_006949.4 | Het | c.971A>G (p.Lys324Arg) | Missense | 0.0408 | 0.0003976 | benign | Tolerated | Neutral | Uncertain significance |
| 184 | 1 | Male | STXBP2 | NM_006949.4 | Het | c.1168G>A (p.Val390Ile) | Missense | 0.0408 | 0.002392 | possibly damaging | Tolerated | Neutral | Uncertain significance |
| 185 | 68 | Male | STXBP2 | NM_006949.4 | Het | c.1210_1210dup (p.Ile404fs) | Frameshift | 0.0408 | NA | NA | NA | NA | Possibly pathogenic |
| 186 | 19 | Male | STXBP2 | NM_006949.4 | Het | c.1289A>G (p.Asn430Ser) | Missense | 0.0408 | NA | benign | Damaging | Neutral | Uncertain significance |
| 187 | 1 | Male | STXBP2 | NM_006949.3 | Het | c.1356+5G>A | Intronic | 0.0817 | 0.005032 | NA | NA | NA | Uncertain significance |
| 188 | 14 | Male | STXBP2 | NM_006949.3 | Het | c.1356+5G>A | Intronic | 0.0817 | 0.005032 | NA | NA | NA | Uncertain significance |
| 189 | 54 | Male | STXBP2 | NM_006949.4 | Het | c.1393C>T (p.Arg465Cys) | Missense | 0.0817 | 0.01172 | probably damaging | Damaging | Deleterious | Uncertain significance |
| 190 | 27 | Male | STXBP2 | NM_006949.4 | Het | c.1393C>T (p.Arg465Cys) | Missense | 0.0817 | 0.01172 | probably damaging | Damaging | Deleterious | Uncertain significance |
| 191 | NA | Female | STXBP2 | NM_006949.3 | Het | c.1412A>G (p.Gln471Arg) | Missense | 0.0408 | NA | possibly damaging | Damaging | Deleterious | Uncertain significance |
| 192 | 40 | Male | STXBP2 | NM_006949.4 | Het | c.1420C>T (p.Arg474Cys) | Missense | 0.0408 | 0.001841 | probably damaging | Damaging | Deleterious | Uncertain significance |
| 193 | 11 | Male | STXBP2 | NM_006949.3 | Het | c.1624G>A (p.Val542Met) | Missense | 0.0408 | NA | benign | Tolerated | Neutral | Uncertain significance |
| 194 | 9 | Male | AP3B1 | NM_003664.5 | Het | c.8G>C (p.Ser3Thr) | Missense | 0.0408 | 0.0003984 | benign | Tolerated | Neutral | Uncertain significance |
| 195 | 46 | Female | AP3B1 | NM_003664.5 | Het | c.31_33dup (p.Gln11dup) | InFrame | 0.0408 | NA | NA | NA | NA | Uncertain significance |
| 196 | 7 | Male | AP3B1 | NM_003664.5 | Het | c.292G>C (p.Val98Leu) | Missense | 0.0408 | NA | possibly damaging | Damaging | Deleterious | Uncertain significance |
| 197 | 32 | Male | AP3B1 | NM_003664.4 | Het | c.311G>T (p.Arg104Leu) | Missense | 0.0408 | NA | probably damaging | Damaging | Deleterious | Uncertain significance |
| 198 | 41 | Female | AP3B1 | NM_003664.4 | Het | c.914A>G (p.Lys305Arg) | Missense | 0.0408 | NA | benign | Tolerated | Deleterious | Uncertain significance |
| 199 | 23 | Male | AP3B1 | NM_003664.4 | Het | c.967T>C (p.Tyr323His) | Missense | 0.0408 | NA | benign | Damaging | Deleterious | Uncertain significance |
| 200 | 4 | Male | AP3B1 | NM_003664.4 | Het | c.1075A>G (p.Thr359Ala) | Missense | 0.0408 | 0.003187 | possibly damaging | Damaging | Deleterious | Uncertain significance |
| 201 | 62 | Male | AP3B1 | NM_003664.5 | Het | c.1243del (p.Ser415fs) | Frameshift | 0.0817 | NA | NA | NA | NA | Possibly pathogenic |
| 202 | 5 | Female | AP3B1 | NM_003664.5 | Het | c.1423C>G (p.Gln475Glu) | Missense | 0.0408 | NA | benign | Tolerated | Neutral | Uncertain significance |
| 203 | 4 | Male | AP3B1 | NM_003664.4 | Het | c.2012C>T (p.Ser671Phe) | Missense | 0.0817 | NA | benign | Damaging | Deleterious | Uncertain significance |
| 204 | 13 | Male | AP3B1 | NM_003664.5 | Het | c.2603C>A (p.Thr868Lys) | Missense | 0.0408 | 0.0007072 | benign | Tolerated | Neutral | Uncertain significance |
| 205 | 64 | Female | AP3B1 | NM_003664.5 | Het | c.2779G>A (p.Gly927Ser) | Missense | 0.0408 | 0.00719 | benign | Damaging | Deleterious | Uncertain significance |
| 206 | 1.4 | Female | AP3B1 | NM_003664.4 | Het | c.3197C>T (p.Ser1066Phe) | Missense | 0.0817 | 0.007072 | benign | Damaging | Deleterious | Uncertain significance |
| 207 | 35 | Male | AP3B1 | NM_003664.4 | Het | c.3197C>T (p.Ser1066Phe) | Missense | 0.0817 | 0.007072 | benign | Damaging | Deleterious | Uncertain significance |
| 208 | 23 | Female | STX11 | NM_003764.4 | Het | c.114C>A (p.Asp38Glu) | Missense | 0.0408 | 0.00199 | benign | Tolerated | Neutral | Uncertain significance |
| 209 | 47 | Male | STX11 | NM_003764.4 | Het | c.374C>T (p.Ala125Val) | Missense | 0.0408 | NA | benign | Tolerated | Neutral | Uncertain significance |
| 210 | 1.2 | Male | STX11 | NM_003764.3 | Het | c.383C>T (p.Ser128Leu) | Missense | 0.0408 | NA | benign | Damaging | Neutral | Uncertain significance |
| 211 | 38 | Male | STX11 | NM_003764.3 | Het | c.463G>A (p.Asp155Asn) | Missense | 0.0408 | 0.001597 | benign | Tolerated | Neutral | Uncertain significance |
| 212 | 5 | Female | STX11 | NM_003764.4 | Het | c.482T>C (p.Ile161Thr) | Missense | 0.0408 | NA | probably damaging | Damaging | Deleterious | Uncertain significance |
| 213 | 4 | Female | STX11 | NM_003764.4 | Het | c.503T>C (p.Met168Thr) | Missense | 0.0408 | NA | benign | Damaging | Neutral | Uncertain significance |
| 214 | 47 | Male | STX11 | NM_003764.4 | Het | c.721A>C (p.Thr241Pro) | Missense | 0.0408 | NA | probably damaging | Tolerated | Neutral | Uncertain significance |
| 215 | 30 | Female | STX11 | NM_003764.4 | Het | c.776C>T (p.Ala259Val) | Missense | 0.0408 | 0.001218 | possibly damaging | Tolerated | Neutral | Uncertain significance |
| 216 | 7 | Male | RAB27A | NM_004580.4 | Het | c.143A>G (p.Glu48Gly) | Missense | 0.0408 | 0.0003986 | probably damaging | Damaging | Deleterious | Uncertain significance |
| 217 | 1 | Male | RAB27A | NM_004580.5 | Het | c.35T>C (p.Phe12Ser) | Missense | 0.0408 | 0.001597 | probably damaging | Damaging | Deleterious | Uncertain significance |
| 218 | 19 | Female | RAB27A | NM_004580.4 | Het | c.560G>A (p.Arg187Gln) | Missense | 0.2042 | 0.0495 | benign | Tolerated | Neutral | Uncertain significance |
| 219 | 19 | Female | RAB27A | NM_004580.4 | Het | c.560G>A (p.Arg187Gln) | Missense | 0.2042 | 0.0495 | benign | Tolerated | Neutral | Uncertain significance |
| 220 | 23 | Male | RAB27A | NM_004580.4 | Het | c.560G>A (p.Arg187Gln) | Missense | 0.2042 | 0.0495 | benign | Tolerated | Neutral | Uncertain significance |
| 221 | 16 | Female | RAB27A | NM_004580.5 | Het | c.614C>A (p.Ala205Asp) | Missense | 0.0408 | NA | benign | Tolerated | Neutral | Uncertain significance |
| 222 | 2 | Male | ITK | NM_005546.3 | Het | c.340A>C (p.Asn114His) | Missense | 0.0408 | NA | possibly damaging | Tolerated | Neutral | Possibly pathogenic |
| 223 | 1.5 | Female | ITK | NM_005546.3 | Het | c.559C>T (p.Pro187Ser) | Missense | 0.0408 | NA | benign | Tolerated | Neutral | Uncertain significance |
| 224 | 59 | Female | XIAP | NM_001167.4 | Het | c.146G>A (p.Arg49Gln) | Missense | 0.0408 | 0.007636 | possibly damaging | Damaging | Deleterious | Uncertain significance |
| 225 | 8 | Female | XIAP | NM_001167.4 | Het | c.295G>C (p.Glu99Gln) | Missense | 0.0408 | NA | benign | Tolerated | Neutral | Uncertain significance |
| 226 | 31 | Female | CD27 | NM_001242.4 | Het | c.779C>T (p.Pro260Leu) | Missense | 0.0408 | 0.0008033 | benign | NA | NA | Uncertain significance |
| 227 | 0.7 | Female | SH2D1A | NM_002351.5 | Het | c.313_315del (p.Lys105del) | InFrame | 0.0408 | NA | NA | NA | NA | Uncertain significance |

*gnomAD v2.1.1 population frequency. ACMG, American College of Medical Genetics and Genomics; Het, heterozygous; Hom, homozygous; Hemi, hemizygous; NA, not available; Polyphen-2, Polymorphism Phenotype v2; PROVEAN, Protein Variation Effect Analyzer; SIFT, Sorting Intolerant From Tolerant.

Table S3. Variants identified in 15 patients with digenic/polygenic variants.

| Patients | Age at testing, year | Sex | Gene | Reference sequence | Zygosity | Variant | Type | Frequency in our chort, % | Frequency in gnomAD*, % | Polyphen2 | SIFT | PROVEAN | ACMG  classification |
| --- | --- | --- | --- | --- | --- | --- | --- | --- | --- | --- | --- | --- | --- |
| 1 | 3 | Male | UNC13D | NM_199242.2 | Het | c.1228A>C (p.Ile410Leu) | Missense | 0.3676 | 0.1159 | benign | Tolerated | Neutral | Uncertain significance |
|  |  |  | LYST | NM_000081.3 | Het | c.452A>G (p.His151Arg) | Missense | 0.1225 | 0.0124 | benign | Damaging | Deleterious | Uncertain significance |
| 2 | 5 | Female | UNC13D | NM_199242.2 | Het | c.2588G>A (p.Gly863Asp) | Missense | 1.5931 | 0.02907 | probably damaging | Damaging | Deleterious | Possibly pathogenic |
|  |  |  | LYST | NM_000081.3 | Het | c.452A>G (p.His151Arg) | Missense | 0.1225 | 0.0124 | benign | Damaging | Deleterious | Uncertain significance |
| 3 | 75 | Female | UNC13D | NM_199242.2 | Het | c.2977C>T (p.Arg993Cys) | Missense | 0.0408 | 0.008988 | possibly damaging | Tolerated | Deleterious | Uncertain significance |
|  |  |  | LYST | NM_000081.3 | Het | c.6079G>C (p.Val2027Leu) | Missense | 0.0817 | 0.006027 | benign | Damaging | Neutral | Uncertain significance |
| 4 | 0.3 | Female | UNC13D | NM_199242.2 | Het | c.1228A>C (p.Ile410Leu) | Missense | 0.3676 | 0.1159 | benign | Tolerated | Neutral | Uncertain significance |
|  |  |  | LYST | NM_000081.3 | Het | c.1544A>G (p.Asp515Gly) | Missense | 0.0408 | NA | probably damaging | Damaging | Deleterious | Uncertain significance |
| 5 | 8.1 | Female | UNC13D | NM_199242.2 | Het | c.1228A>C (p.Ile410Leu) | Missense | 0.3676 | 0.1159 | benign | Tolerated | Neutral | Uncertain significance |
|  |  |  | LYST | NM_000081.3 | Het | c.368A>G (p.His123Arg) | Missense | 0.2451 | 0.08393 | benign | Tolerated | Neutral | Uncertain significance |
|  |  |  | RAB27A | NM_004580.4 | Het | c.560G>A (p.Arg187Gln) | Missense | 0.2042 | 0.0495 | benign | Tolerated | Neutral | Uncertain significance |
| 6 | 58 | Female | UNC13D | NM_199242.3 | Het | c.2756C>T (p.Ser919Phe) | Missense | 0.1225 | 0.004278 | possibly damaging | Tolerated | Neutral | Uncertain significance |
|  |  |  | AP3B1 | NM_003664.5 | Het | c.1243del (p.Ser415fs) | Frameshift | 0.0817 | NA | NA | NA | NA | Possibly pathogenic |
| 7 | 1 | Female | UNC13D | NM_199242.2 | Het | c.2709+1G>A | Intronic | 0.2042 | NA | NA | NA | NA | Pathogenic |
|  |  |  | AP3B1 | NM_003664.4 | Het | c.2012C>T (p.Ser671Phe) | Missense | 0.0817 | NA | benign | Damaging | Deleterious | Uncertain significance |
| 8 | 17 | Female | UNC13D | NM_199242.2 | Het | c.1298G>A (p.Arg433Lys) | Missense | 0.0408 | 0.001197 | probably damaging | Tolerated | Neutral | Uncertain significance |
|  |  |  | RAB27A | NM_004580.4 | Het | c.240-1G>A | Intronic | 0.0408 | NA | NA | NA | NA | Possibly pathogenic |
| 9 | 26 | Female | UNC13D | NM_199242.2 | Het | c.797G>T (p.Arg266Leu) | Missense | 0.0408 | 0.0003994 | possibly damaging | Damaging | Deleterious | Uncertain significance |
|  |  |  | RAB27A | NM_004580.4 | Het | c.11G>T (p.Gly4Val) | Missense | 0.0408 | 0.003211 | benign | Tolerated | Neutral | Uncertain significance |
| 10 | 2.1 | Female | LYST | NM_000081.3 | Het | c.6775G>A (p.Val2259Ile) | Missense | 0.1225 | 0.006383 | benign | Tolerated | Neutral | Uncertain significance |
|  |  |  | STXBP2 | NM_006949.3 | Het | c.1597C>T (p.Arg533Trp) | Missense | 0.0408 | 0.004836 | probably damaging | Damaging | Deleterious | Uncertain significance |
| 11 | 19 | Female | LYST | NM_000081.3 | Het | c.6735C>G (p.Ser2245Arg) | Missense | 0.0817 | NA | possibly damaging | Damaging | Neutral | Uncertain significance |
|  |  |  | RAB27A | NM_004580.4 | Het | c.560G>A (p.Arg187Gln) | Missense | 0.2042 | 0.0495 | benign | Tolerated | Neutral | Uncertain significance |
| 12 | 57 | Male | LYST | NM_000081.3 | Het | c.8351C>G (p.Ser2784Cys) | Missense | 0.0817 | 0.001196 | benign | Damaging | Deleterious | Uncertain significance |
|  |  |  | RAB27A | NM_004580.4 | Het | c.551G>A (p.Arg184Gln) | Missense | 0.0408 | 0.01202 | benign | Damaging | Deleterious | Uncertain significance |
| 13 | 1 | Female | LYST | NM_000081.3 | Het | c.3730G>C (p.Glu1244Gln) | Missense | 0.0408 | NA | benign | Tolerated | Neutral | Uncertain significance |
|  |  |  | STXBP2 | NM_006949.3 | Het | c.974A>G (p.Asp325Gly) | Missense | 0.0408 | 0.0007953 | probably damaging | Tolerated | Deleterious | Uncertain significance |
| 14 | 37 | Male | LYST | NM_000081.4 | Het | c.8418G>C (p.Leu2806Phe) | Missense | 0.0408 | NA | possibly damaging | Damaging | Deleterious | Uncertain significance |
|  |  |  | STXBP2 | NM_006949.4 | Het | c.1214G>A (p.Arg405Gln) | Missense | 0.0817 | 0.001597 | probably damaging | Damaging | Deleterious | Possibly pathogenic |
| 15 | 48 | Female | LYST | NM_000081.4 | Het | c.5578A>G (p.Met1860Val) | Missense | 0.0408 | 0.00142 | benign | Damaging | Neutral | Uncertain significance |
|  |  |  | STX11 | NM_003764.4 | Het | c.338C>G (p.Ala113Gly) | Missense | 0.0408 | 0.000832 | benign | Damaging | Neutral | Uncertain significance |

*gnomAD v2.1.1 population frequency. ACMG, American College of Medical Genetics and Genomics; Het, heterozygous; Hom, homozygous; Hemi, hemizygous; NA, not available; Polyphen-2, Polymorphism Phenotype v2; PROVEAN, Protein Variation Effect Analyzer; SIFT, Sorting Intolerant From Tolerant.

Table S4. Twelve genes that are tested in targeted generation sequencing panel.

| **Symbol**  **(Transcript)** | **Gene** **OMIM** | **Associated** **OMIM** **Disease** | **Inheritance** |
| --- | --- | --- | --- |
| AP3B1  (NM_003664) | 603401 | Hermansky-Pudlak syndrome 2 | AR |
| CD27  (NM_001242) | 186711 | Lymphoproliferative syndrome 2 | AR |
| ITK  (NM_005546.3) | 186973 | Lymphoproliferative syndrome 1 | AR |
| LYST  (NM_000081) | 606897 | Chediak-Higashi syndrome | AR |
| MAGT1  (NM_032121) | 300715 | Immunodeficiency, X-linked, with magnesium defect, Epstein-Barr virus infection and neoplasia | XL |
| PRF1  (NM_001083116) | 170280 | Hemophagocytic lymphohistiocytosis, familial, 2; Aplastic anemia; Lymphoma, non-Hodgkin | AR |
| RAB27A  (NM_004580) | 603868 | Griscelli syndrome, type 2 | AR |
| SH2D1A  (NM_002351) | 300490 | Lymphoproliferative syndrome, X-linked, 1 | XL |
| STX11  (NM_003764) | 605014 | Hemophagocytic lymphohistiocytosis, familial, 4 | AR |
| STXBP2  (NM_006949) | 601717 | Hemophagocytic lymphohistiocytosis, familial, 5 | AR |
| UNC13D  (NM_199242) | 608897 | Hemophagocytic lymphohistiocytosis, familial, 3 | AR |
| XIAP  (NM_001167) | 300079 | Lymphoproliferative syndrome, X-linked, 2 | XL |

AD autosomal dominant, AR autosomal recessive, XL X-Linked
